# Seismic characteristics and AVO response for non-uniform Miocene reservoirs in offshore eastern Mediterranean region, Egypt

**DOI:** 10.1038/s41598-023-35718-z

**Published:** 2023-06-01

**Authors:** Ahmed S. Abu El-Ata, Nader H. El-Gendy, Adly H. El-Nikhely, Samir M. Raslan, Mahmoud S. El-Oribi, Moataz Kh. Barakat

**Affiliations:** 1 Geophysics Department, Faculty of Science, Ain Shams University, Cairo, Egypt; 2 Geology Department, Faculty of Science, Tanta University, Tanta, 31527 Egypt; 3 Physics Department, Faculty of Science, Alexandria University, Alexandria, Egypt; 4 Vice Chairman for Agreements and Exploration/EGAS, Cairo, Egypt; 5 Dana Gas Company, Plot No 188, City Center, Fifth Settlement, New Cairo, Egypt

**Keywords:** Environmental sciences, Energy science and technology

## Abstract

The Eastern Mediterranean region, extending from the Offshore Nile Delta Cone of Egypt to the Levant Basin, is a confirmed hydrocarbon-rich territory with several giant gas discoveries. Numerous gas fields have been discovered in the Miocene reservoirs within the Nile Delta Cone, and the Levant Basin. The Miocene sedimentary sequences in this region are extremely heterogeneous, consisting mainly of turbiditic slope deposits, channels, and basin floor fans that were capped by evaporites formed during the Messinian Salinity Crisis. As a result, the seismic characteristics and interpreted properties of this heterogeneous section are ambiguous. The study area is located in the Offshore North Sinai Basin, where a thick Early Miocene section was deposited midway between the Nile Delta province, which includes the El-Fayrouz discovery, and the Levant Basin, which includes Tamar, Tanin, and several other discoveries. This study uses quantitative seismic interpretations methods, such as amplitude variations with offset and fluid replacement modeling, to assess the seismic acoustic impedance trend with depth. Also, determine the seismic amplitude response for the brine and gas sands reservoir of the Early and Late Miocene section to link the unexplored study area within the North Sinai Offshore Basin with the explored Nile Delta and Levant Basins. In addition to evaluate direct hydrocarbon indicator (DHI) of the dimming seismic amplitude that is compatible with the structure’s last closed contour of the Syrian Arc anticline of the Early Miocene reservoirs (EMT-1 prospect). Different vintages of 2D and 3D seismic data, six wells, and various published data were used in this study. The quantitative interpretation shows the pitfalls of the acoustic impedance trend and seismic response dependency on depth for gas and brine sand, which led to the drilling of the EMT-1 dry well. Also, the fluid replacement, P-wave velocity (Vp), and density (ρ) modeling confirmed that the seismic dimming amplitude was due to a seismic processing artifact, which was corrected by readjusting the overburden Messinian salt processing velocity model. This research concludes that the seismic quantitative interpretations are successfully used to assess the acoustic impedance versus depth and understand DHI pitfalls, as well as the processing workflow that could enhance the seismic image.

## Introduction

The East Mediterranean Miocene section has been well explored within the Nile Delta Basin (Abu Madi, E. Nile Delta, Balsam, Faraskur, Temsah, Raven, and Rahamat fields)^1–4^ and the Levant Basin (Leviathan, Tamar, Aphrodite, Tanin, and Karish fields)^2,5,6^. In the past decades, most of the exploration companies’ activities concentrated on the Late Miocene and Oligocene reservoirs within the Nile Delta and the Early Miocene within the Levant Basin. Within the Eastern Mediterranean region, more than 80 TCF (Trillion Cubic Feet) have been discovered to date within the On /Offshore parts of the Nile Delta Basin including more than 40 TCF in the Levant Basin^6,7^. Many areas continue to hold exploration promise, with the key to unlocking this potential being a better characterization of the seismic response to gas. This is especially true for high cost deep marine wells targeting Miocene reservoirs.

**Figure 1:**
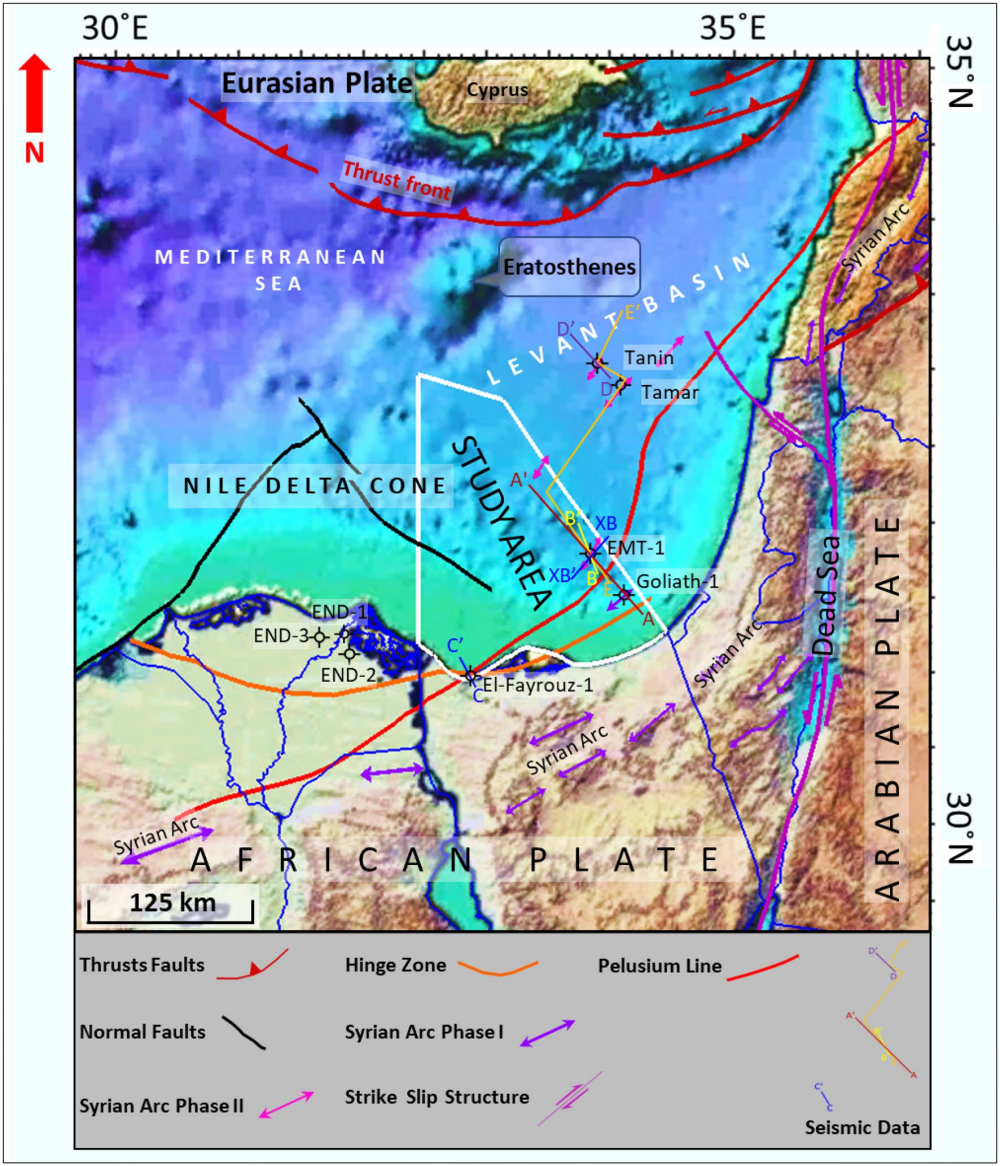
East Mediterranean Region colored terrain map with the possible structural framework. Created in Dana Gas Egypt using Petrel 2020 software, https://www.software.slb.com/products/petrel.

**Figure 2:**
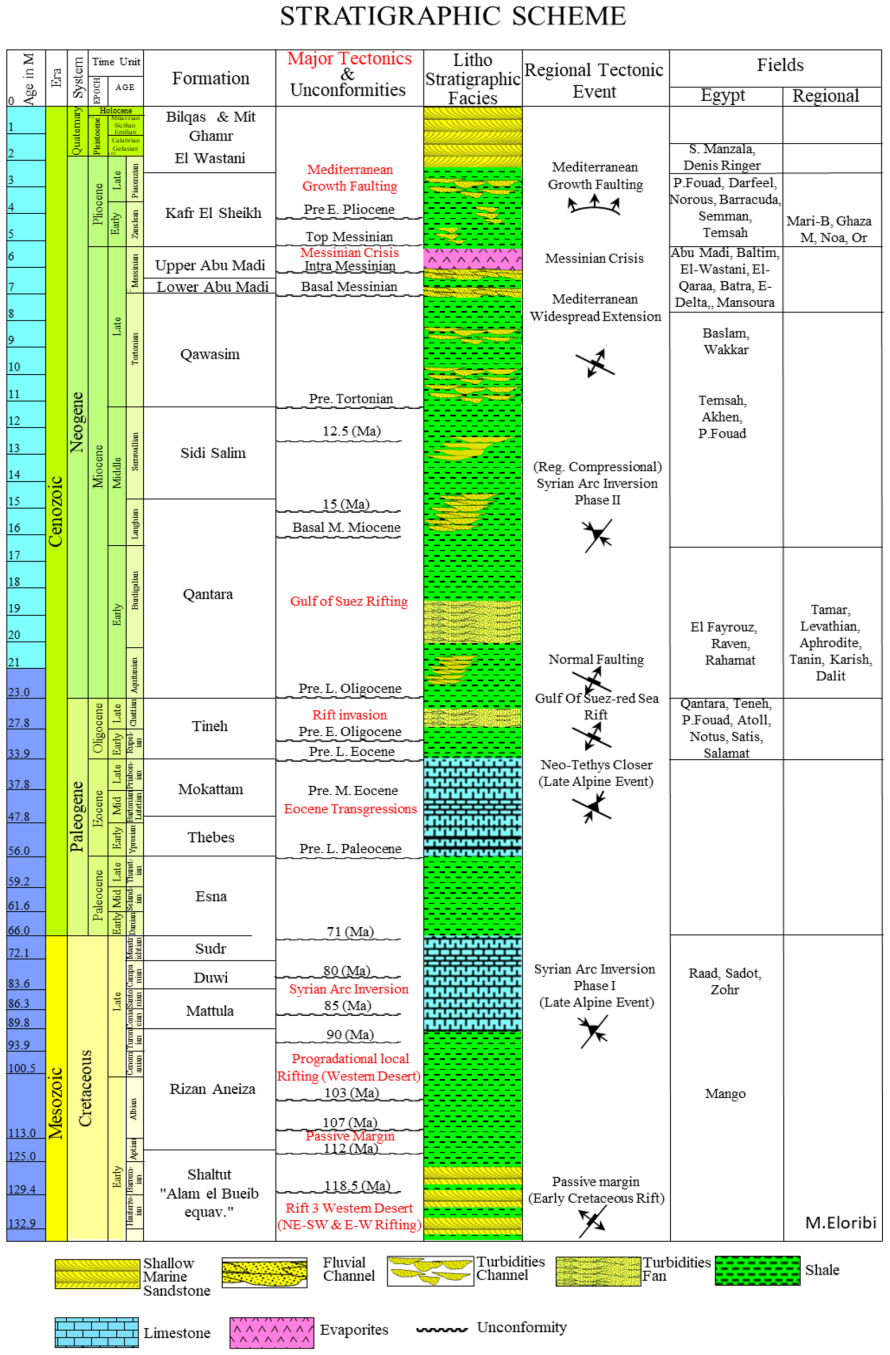
Mediterranean offshore stratigraphic column scheme (after^7^) with regional tectonic events and facies of areas of interest.

**Figure 3:**
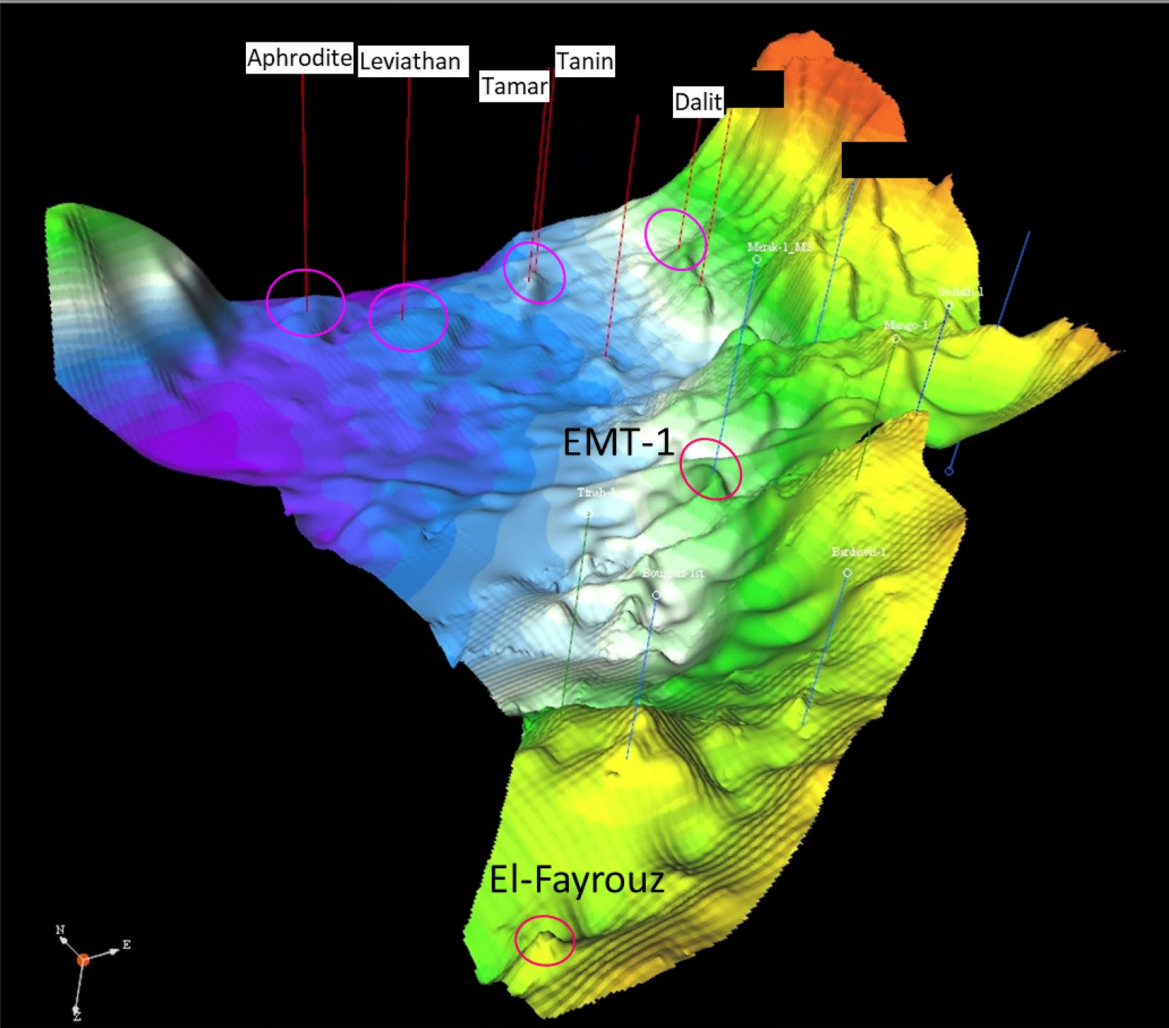
Top of Early Miocene 3D view structural map shows the EMT-1 target structure of this study, Nile Delta discovery (El-Fayrouz), and Levant Basin discoveries (Aphrodite, Leviathan, Tamar, Tanin, and Dalit). Created in Dana Gas Egypt using software kingdom 2015, https://kingdom.ihs.com/.

The study area (Fig. 1) is located within unexplored acreage at the margins of both the Nile Delta Cone and Levant Basins, where the Early Miocene anticlinal structure of the Syrian Arc System Phase II is delineated, similar to the Levant Tamar discoveries as in the stratigraphic column shown (Fig. 2). The Syrian Arc System Phase II started within the end of the Early Miocene and during the Mid Miocene due to a regional compressional within the North Africa and Eastern Mediterranean (Fig. 2). A 3D-view of Early Miocene depth structural map shows the study area and the EMT-1 structure as the first well to be drilled within the Offshore North Sinai Basin (Fig. 3).

Several studies of the Eastern Mediterranean region are mainly focused on the Oligocene and Miocene sections of the Nile Delta Basin in relation to the tectonic evolution of the Eastern Mediterranean Basin and its significance for the hydrocarbon prospectivity of the Nile Delta deep-water area^3,7–9^. The Miocene sequence stratigraphic framework^10,11^, particularly the Late Miocene section, and the petrophysical evaluation of the Oligocene reservoirs in the East Mediterranean Offshore Area and the Nile Delta of Egypt^12–14^ have also been examined.

Other studies focused on the Oligo-Miocene deep-water system of the Levant Basin^6,15^. The giant Tamar gas field established the viability of subsalt Miocene basin floor fan sand as a gas play in the Levant Basin^5,6,14^ with the Oligo–Miocene closure of the Tethys Ocean and evolution of the proto‑Mediterranean Sea^16^. The Miocene section is now considered a prolific hydrocarbon-bearing interval within the Eastern Mediterranean Sea and the Nile Delta and Levant Basin. The Early Miocene Target well (EMT-1) was the first well to be drilled to test the Syrian Arc System Phase II anticline structure within the study area in the Offshore North Sinai Basin (Fig. 3).

Seismic (AVO) modeling forms the basis for understanding the systematic AVO seismic responses and signatures with depth^17^ and enables prediction of reservoir characteristics away from well control^18,19^. The study area has not yet been explored, with the nearest wells that penetrate the section of interest being El Fayrouz-1 located 128 km to the west within the Nile Delta province and the Tamar wells located 135 km to the east toward the Levant Basin (Fig. 3). For predictions away of the wells control, lateral and vertical variations can be inferred from the rock physics studies^20,21^ that include the impact of pore fluids, lithologies, and depths on the AVO signatures^22^.

During AVO analysis, it is necessary to understand the seismic amplitude response and acoustic impedance (AI) behavior of the shales and sands as a function compaction versus depth^23^. The AVO analysis and AI trends can also be used to predict the expected seismic signatures of sand-shale interfaces as a function of depth and to identify anomalous lithologies or diagenetic zones.

**Figure 4:**
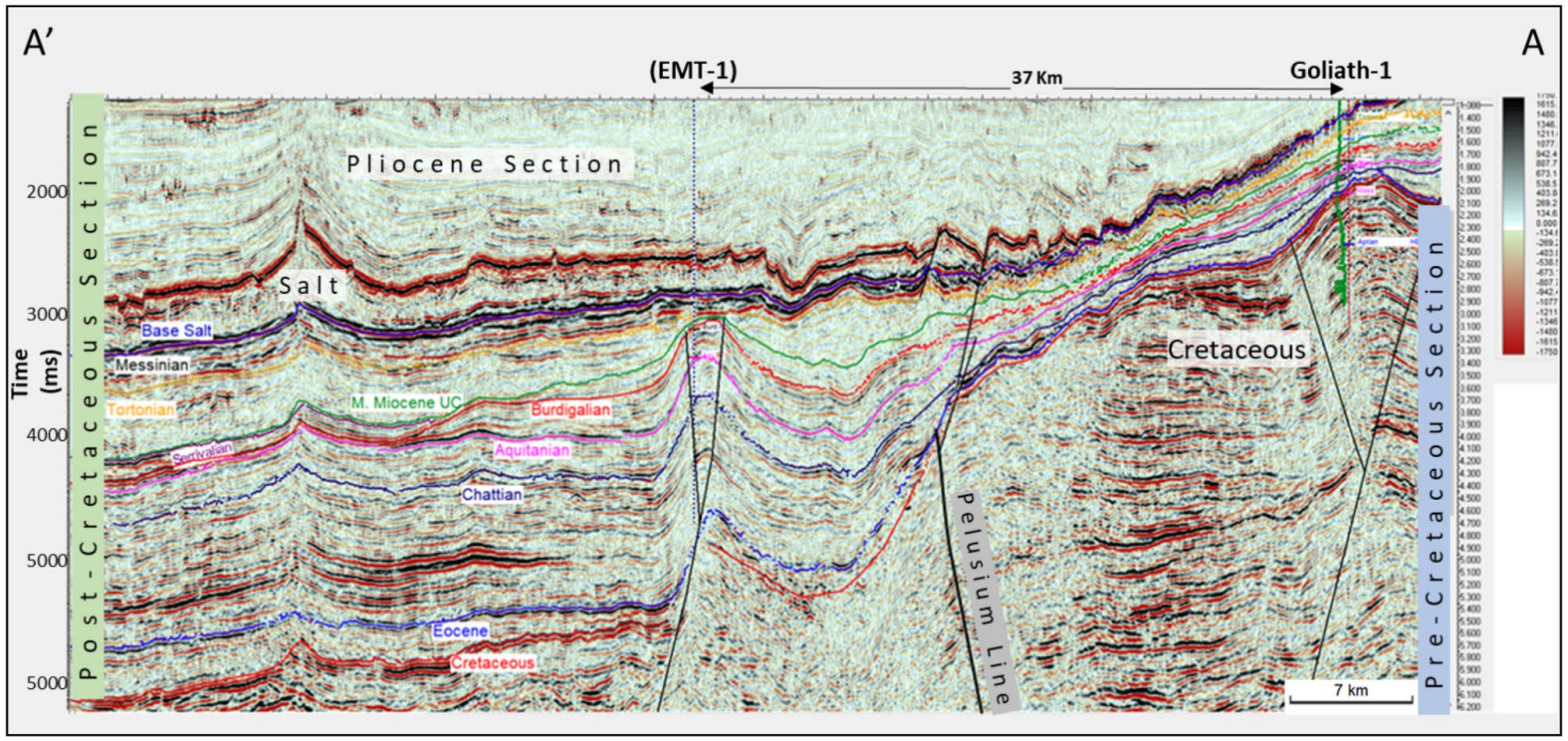
AA’ NW–SE seismic profile as shown in Fig. 1, passing by the study area shows Goliath-1 Cretaceous Syrian Arc Phase I structure, and EMT-1 Miocene structure developed down dip of the Pelusium Line during Mid- Miocene Syrian Arc Phase II.

Minimal data are available for Early Miocene reservoirs within the study area, which are the main focus of this study. So, a detailed seismic amplitude versus offset (AVO) modeling study was carried out for the different Miocene-age sections from several fields within the Nile Delta and Levant Basins to validate the approach using the known nearby discoveries and to assess the applicability for the Early Miocene Syrian Arch Phase II anticlinal structure within the study area. This study is focused on one of the anticlinal structures at the margin between the Nile Delta Cone and Levant Basin (Figs. 3 and 4). The objective of this study is using the quantitative interpretations to evaluate and assess the AVO modeling and AI responses to describe the seismic signature characteristics with depth for the Early Miocene reservoir section within the Offshore North Sinai Basin (the study area) and to do so within the regional context of the Nile Delta and Levant Basins across the Eastern Mediterranean region. The quantitative interpretation was successfully used to assess the acoustic impedance versus depth and to improve the processing workflow that could enhance the seismic image.

## Geologic setting

The Eastern Mediterranean region, which include the Nile Delta cone, North Sinai Basin and the Levant Basins, is located near the conjunction of the African (Nubian Plate & Sinai Plate) -Arabian-Eurasian Plates (Fig. 1) and was formed by the Permian, Triassic, and Early Jurassic pulses of rifting that shaped the African continental margin^24–30^. It is the remnant of the large Neo-Tethyan Ocean that opened between several fragments of the Pangea supercontinent in Early Mesozoic times^24,27,31,32^. The North Sinai basin, as a part of the Eastern Mediterranean region, is bounded from the south and by the Syrian Arc elevated topographic features at the onshore parts of Northern Egypt and the Levant (Fig. 1), marking the southern and Eastern most limit of the coastal plains. These elevated features represent inversion folds that extend from Onshore Syria to the North of Sinai Peninsula and extend to the subsurface of the Northern Western Desert of Egypt^33–36^. These inverted folds are known as the ‘‘Syrian Arc System’’ (Fig. 1)^2,3,37,38^.

The Syrian Arc structural folding and chronological similarity within the onshore and Offshore parts imply the presence of a broad deformation extending land-ward from Syria to Sinai and Western Desert of Egypt, and seaward to the Eratosthenes Seamount and Cyprus^7,37,39^. The Syrian Arc System deformational zone took place in several pulses, starting in the Santonian (Syrian Arc Phase I ~ 85 Ma) and continued until the Early to Mid-Miocene (Syrian Arc Phase II ~ 16 Ma)^7,38,40^. Syrian Arc Phase II anticlinal structures in the Levant Basin have been successfully tested in the Early Miocene-age discoveries^41^.

The Basin is bounded to the north by the subduction-related thrust fault belts associated with the convergence between the African and Eurasian Plates (Fig. 1). These thrust fault belts are known as ‘‘the Alpine-Thrust Front’’^42–45^. Eratosthenes is a paleo-high, with a present-day bathymetric feature covered by a continental crust fragment that detached from the African continental margin during the Triassic Early rift phase^46,47^.

During the Miocene, some major tectonic plate configuration changes took place, which affected North Sinai Basin and the whole Eastern Mediterranean region, with subduction of the African Plate beneath the Eurasian Plate south of Cyprus at the north. On the other hand, the Dead Sea transform separating the African Plate from the Arabian Plate at the east (Fig. 1)^27,28,48–53^.

The Miocene sedimentary section consists of different sedimentary environments comprised mainly of turbidite slope deposits, basin floor fans, and channel sands that are capped by the Messinian Salinity Crisis evaporites (Fig. 2). During the Early Miocene, the main depocenter of the sediment accumulation was located eastward of the study area in the Levant Basin^54^. The Early Miocene section thickens to the East and Northeast while the Middle to Late Miocene section thickens Northwest ward^7^. A broad folding (Syrian Arc Phase II) in the deep basin occurred in the Early Miocene and continued through the Middle Miocene^55–57^. The folding activity in the entire Mediterranean Sea declined in the Late Miocene when the sea closed during the Messinian Salinity Crisis^58^. During the Early Miocene (Aquitanian to Langhian), the main clastic sandstones and interbedded shale were deposited in the Nile Delta Basin (e.g., Raven, Rahamat, Hout, and El Fayrouz wells) and Levant Basin (e.g., Tamar well)^8,59^ (Fig. 2).

The Middle Miocene Serravallian to Tortonian sequence was dominated by clastic sandstone turbidites throughout the entire basin (e.g., Temsah field) at the Eastern Nile Delta, probably thinning toward the Levant Basin^7,52^. The Late Tortonian marked the second structural growth period during the Neogene with the onset of the activity of the Dead Sea Transform Faults (Figs. 1 and 2)^49,60^.

The Late Miocene Messinian period marked a major sea-level drop and desiccation of the Mediterranean Sea and deposition of the Messinian evaporites ^61,62^. Despite the evaporitic nature of the Messinian strata, a massive fluvial canyon exists during this time, forming the Abu Madi-Baltim fluvial canyon trend within the Nile Delta Cone^61^ (Fig. 2). As inferred from seismic data, the Messinian Salt reached a thickness of ~ 2 km in the Levantine Basin with the anhydrite in the proximal part being much thinner^63^.

## Data and methods

Five wells were selected to achieve this study in addition to the EMT-1 well, two wells (El Fyarouz-1, Tanin-1) tested the Early Miocene section and used in the pre-drill study forward model in order to evaluate the EMT-1 well structure as the main focus of this study (Figs. 1 and 3). El Fyarouz-1 well is 127 km away from EMT-1 to the West, while Tanin-1 well is 147 km to the East. The other three wells within the Nile Delta Cone (END-1, END-2, END-3) tested Late Miocene section, used to assess the seismic response and AI behavior versus depth. (Fig. 2). Moreover, Pre-stack depth migration (PSDM) seismic data used to evaluate the EMT-1 well structure and a regional 2D seismic data is used to tie the study area to the Levant Basin (Fig. 1).

**Figure 5:**
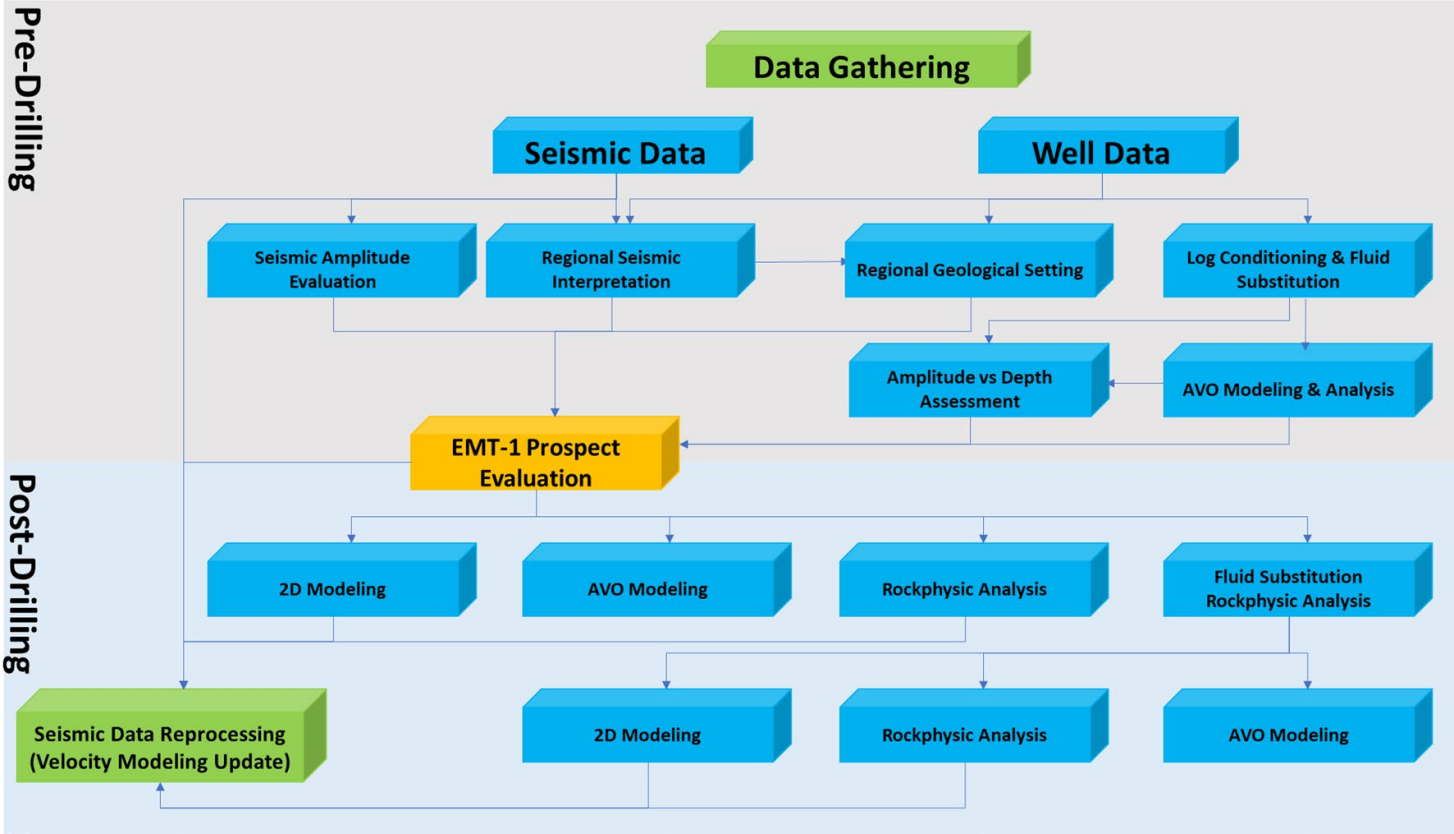
Flow Diagram of the Methodology in the study shows the pre-drilling and post-drilling of the EMT-1 Well.

The 3D seismic data was acquired using the recent broadband acquisition approach with goal to improve signal to noise ratio at the target level. Data was acquired with wide tow but with the narrow azimuth. Processing sequence consists of standard steps: Noise attenuation, De-ghosting, Surface Multiple Attenuation, Regularization, Migration and Post Migration Data Conditioning. Figure 5 shows the flow diagram that summarizes the methodology in the study and shows the pre-drilling and post-drilling workflow of the EMT-1 well.

In seismic reflection, the amplitude character generally varies with offsets (AVO) or the change of the incidence angle (AVA). Of the two, AVO assessment allows better determination and evaluation of reservoir rock properties and fluid content, depending on the velocity, density, and Poisson’s ratio contrast^19^. Knott^64^ and Zoeppritz^65^ developed the theoretical basis for the AVO theory. They developed the equations for plane-wave reflection amplitudes as a function of the incidence angle. Bortfeld^66^ simplified Zoeppritz’s equations to understand how the reflection amplitudes vary or depend on the incidence angle and physical parameters. AVO analysis and interpretation can be used to detect laterally the changes in the elastic properties of layers, such as the changes in seismic velocities and Poisson’s ratios, which may indicate a change in the pore fluid properties^67^.

The reflections from various subsurface interfaces exhibit a wide range of AVO characteristics that depend mainly upon the rock types and pore fluid contents^68^. The reflection coefficient curves classified by Rutherford and Williams^69^ have become the industry standard. It is respectively associated with the 1970s classifications of the bright spot, dim out, and phase reversal. The reflection coefficient curve slope is negative for all the classes within Rutherford and Williams’s classification. The amplitude decreases with the angle of incidence for Class I gas saturated AVO anomalies and increases with the angle of incidence for classes 2 and 3. A certain gas saturated AVO anomaly can have slowly decreasing amplitudes with the offset named Class IV by^70^.

**Figure 6:**
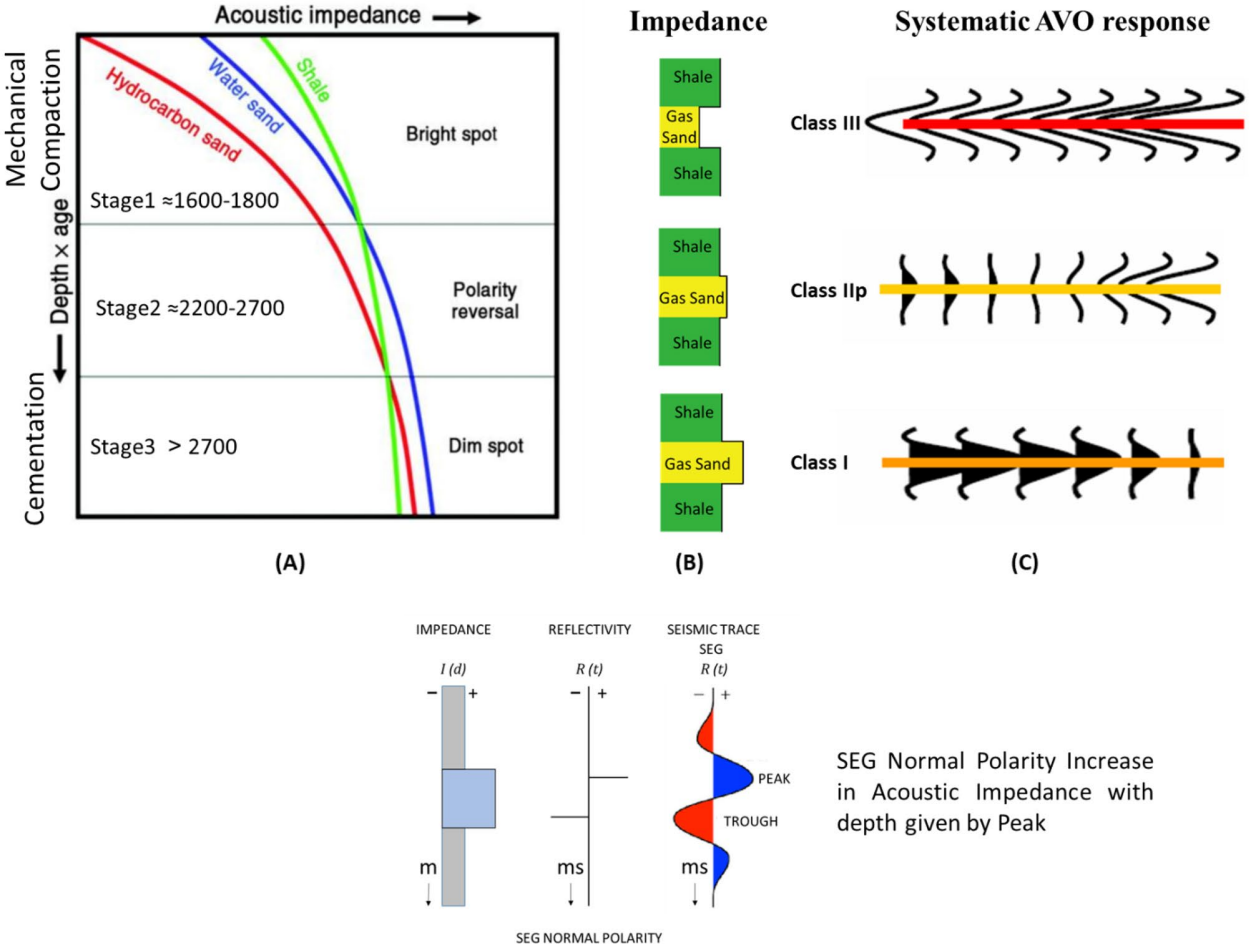
(**A**) Expected acoustic impedance trends and responses to hydrocarbons ^74^, (**B**) Expected acoustic impedance model for the gas sand, and (**C**) the possible AVO seismic signatures with depth (Age).

There are several hypotheses regarding the change in AVO response and amplitude character with depth and how the behavior of the reflection coefficient changes. The general consensus is that the shallow gas layers are affected by the mechanical compaction (Fig. 6A) and exhibit a typical Class III (Fig. 6C) interface with a high amplitude bright spot^23^. The deeper sections (down to ~ 2.4 km below the surface) exhibit typical Class II behavior of little impedance contrast between the shale and the underlying sand layers and the deepest layers are affected by the cementation (deeper than ~ 2.7 km below the surface) and behave like Class I, which has the highest impedance sand, causing a decrease in the amplitude with offset. The effect of gas on the reflection coefficient is also low in the deep sedimentary section due to overburden pressure and rock cementation (reducing the petrophysical parameters)^23,71–73^ (Fig. 6)^74^.

Studying the AVO and AI behavior for a certain reservoir with different fluids type (wet and gas cases), fluid replacement (fluid substitution), and using Gassmann’s equation^75^ on the reservoir well data to change the fluid type and the saturation to assess the AVO and AI behavior for the different conditions. Also, it is possible to compare the various pore fluid conditions with the seismic data by creating a synthetic seismograph using Zoeppritz's equations or one of its approximations.

There are three stages that could control the AI trends and the seismic signatures of sand-shale interfaces as a function of depth, which describe the anomalous lithologies or diagenetic zones^23,76^ (Fig. 6A).

- *Stage 1* During sediment deposition, shallow unconsolidated sand (1600–1800 m) depending on the degree of mechanical compaction (Bright Spot).
- *Stage 2* During burial, phase reversal (2200–2700 m) is due to cementation (Mechanical compaction; overpressure of approximately 1900–2000 m).
- *Stage 3* Sand is cemented, and the shale fluid is being squeezed out resulting in diagenetic zones (Dimout Amplitude, Dim Spot).

These three stages will be assessed in this research for the Early and Late Miocene reservoirs.

## Results

**Figure 7:**
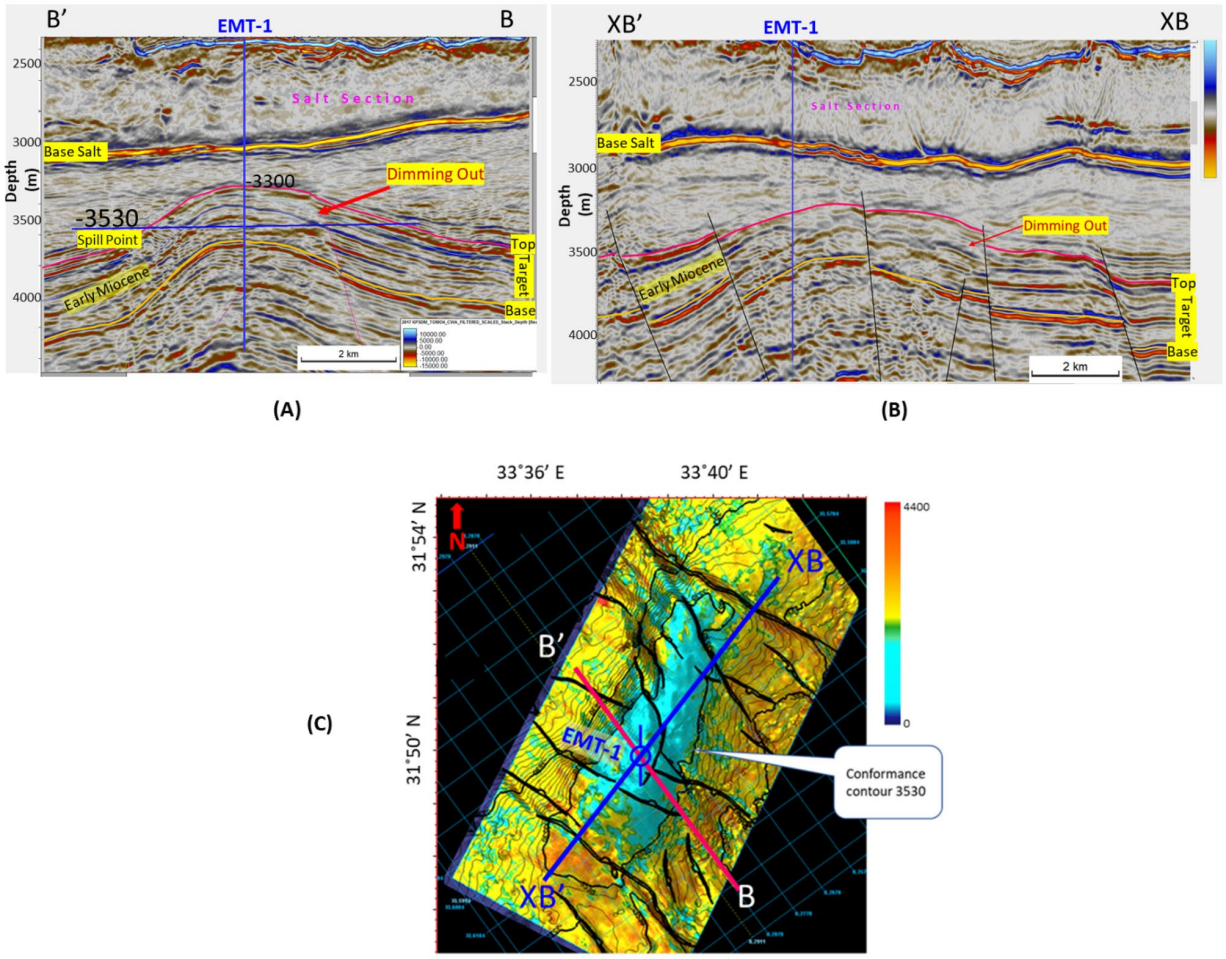
(**A**) BB’ seismic section as the location shown in Fig. 1, and (**B**) the perpendicular seismic section XBXB’, show EMT-1 Early Miocene structure, where the top target characterized by dim amplitude above a certain flat event at 3530 m which matches the spill point of the structure, (**C**) Amplitude map overlayed by structure contour lines, which shows the good match of the dim amplitude with the structure spill. Created in Dana Gas Egypt using Petrel 2020 software, https://www.software.slb.com/products/petrel.

The Early Miocene Target (EMT-1) is the first well to be drilled in the Syrian Arc Phase II structure within the study area in the Offshore North Sinai Basin. The structure took place in the Early Miocene and continued through the Middle Miocene on the down-dip side of the Pelusium Line and the older Cretaceous Syrian Arc Phase I targeted by the Goliath-1 well, as more than 1000 m of sediments accumulated in the study area beneath the Messinian Salt in a localized depocenter (Fig. 4). The 3D seismic interpretation and amplitude map of the Early Miocene section (Burdigalian and Aquitanian) show a dim seismic amplitude, partially controlled by the structure lowest closed contour (the spill of the structure) (Fig. 7C), and coincidentally leveled with a flat event at -3530 m TVDSS (True Vertical Depth Sub-Sea) (Fig. 7). According to the expected seismic amplitude response with depth (Fig. 6), the reservoir character response at the top target depth of -3300 m TVDSS should exhibit a dim out of the seismic amplitude since the reservoir should follow the stage-3 AI behavior as the sand is cemented and the shale is being squeezed out resulting in diagenetic zones (Fig. 6A). So, the gas sand will have a higher in AI than the shale above, which is represented by dimout amplitude (Fig. 7C).

Based on the mismatch between the seismic amplitude response with depth (DHI, dim out amplitude) and the unexpected result of EMT-1 drilling, the next logical step was to examine the seismic response associated with Miocene discoveries in nearby basins.

### Seismic signatures and responses with depth

There is ample data available for Late Miocene onshore discoveries within the East Nile Delta (END) area that can be used to assess seismic signature responses and AI behavior with depth. There are three Late Miocene wells (END-1, END-2, and END-3) (Fig. 1) that demonstrate both expected and unexpected AI behavior with depth.

**Figure 8:**
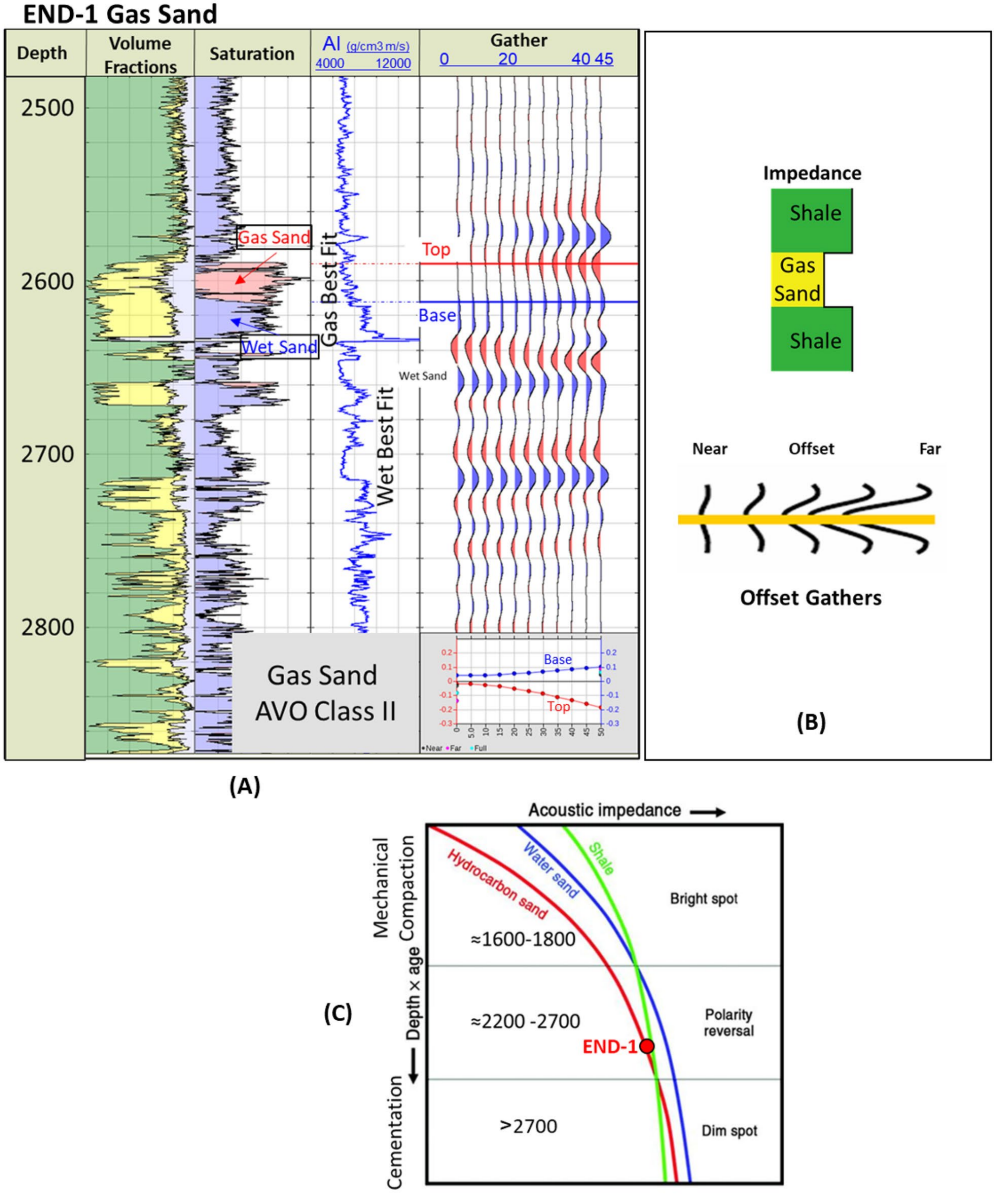
END-1 well Late Miocene gas sand, (**A**) Synthetic gather analysis shows AVO class II gas sand, (**B**) Schematic form shows the impedance of the gas sand and offset gathers of AVO class II, (**C**) Acoustic impedance trends model of END-1 gas sand that plotted within the polarity reversal zone.

The END-1 well penetrates the Late Miocene Messinian–Upper Abu Madi channel gas sand at a depth of 2585 m (Fig. 8A). The AI of the gas sand is slightly lower than that of the overburden shale (Fig. 8A (AI log) and B). The END-1 well synthetic offset gathers, and the schematic offset gathers indicate that the AVO behavior is gas sand Class II, which starts with a dim negative amplitude (Trough) at the near offsets (or angles), and this negative amplitude increases with the far offsets (angles) (Fig. 8A and B). The AI trend for the END-1 well shows that the Upper Abu Madi hydrocarbon-bearing sand exists within the polarity reversal zone, which extends from 2200 to 2700 m (Fig. 8C).

**Figure 9:**
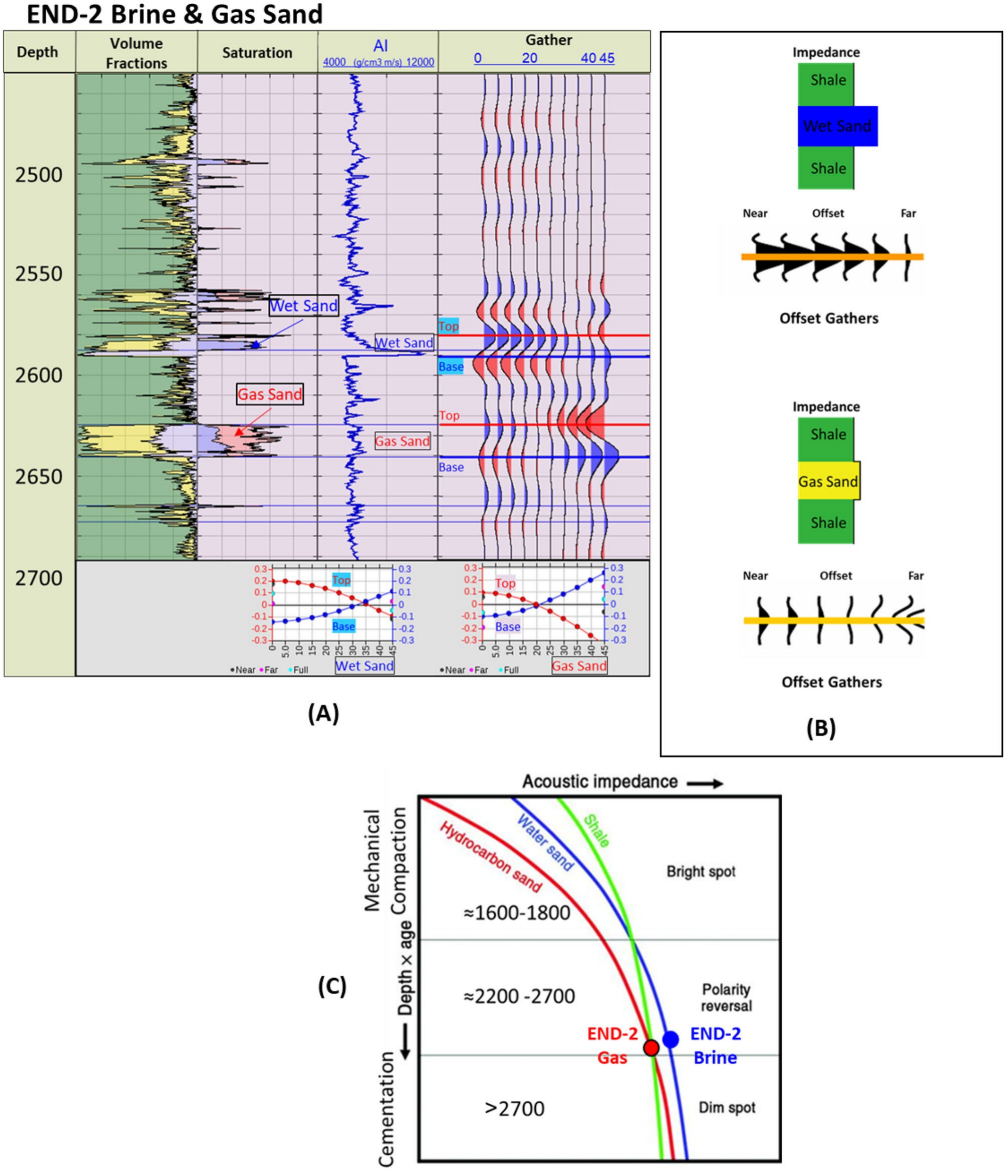
END-2 well Late Miocene wet & gas sand, (**A**) Synthetic gather analysis shows AVO classes I & IIp respectively, (**B**) Schematic form shows the impedance and offset gathers of wet & gas sand, (**C**) Acoustic impedance trends model of END-1 wet & gas sand that plotted within the polarity reversal zone.

The END-2 well (Fig. 9) penetrates the Late Miocene Messinian–Upper Abu Madi channel water-bearing sand (wet sand) at a depth of 2580 m. The AI of the wet sand is significantly higher than the overburden shale, while the Lower Abu Madi gas sand at depth 2630 m is represented by AI that is slightly higher than the overburden shale (Fig. 9A (AI log) and B). The Upper Abu Madi wet sand synthetic offset gathers indicate that the AVO behavior is brine (wet) sand Class I, starting with a bright positive amplitude (Peak) at the near offsets which decreases with the far offsets (Fig. 9A and B). The Lower Abu Madi gas sand is behaving as AVO Class IIp, which starts with a slight dim positive amplitude (Peak) at the near offsets and then decreases in amplitude with the offset until it changes to a trough at an angle of 20 degrees, where the amplitude becoming more negative at the far offsets (Fig. 9A and B). The AI trends for the END-2 well show that the Abu Madi brine and gas sands exist within the polarity reversal zone, which extends from 2200 to 2700 m (Fig. 9C).

**Figure 10:**
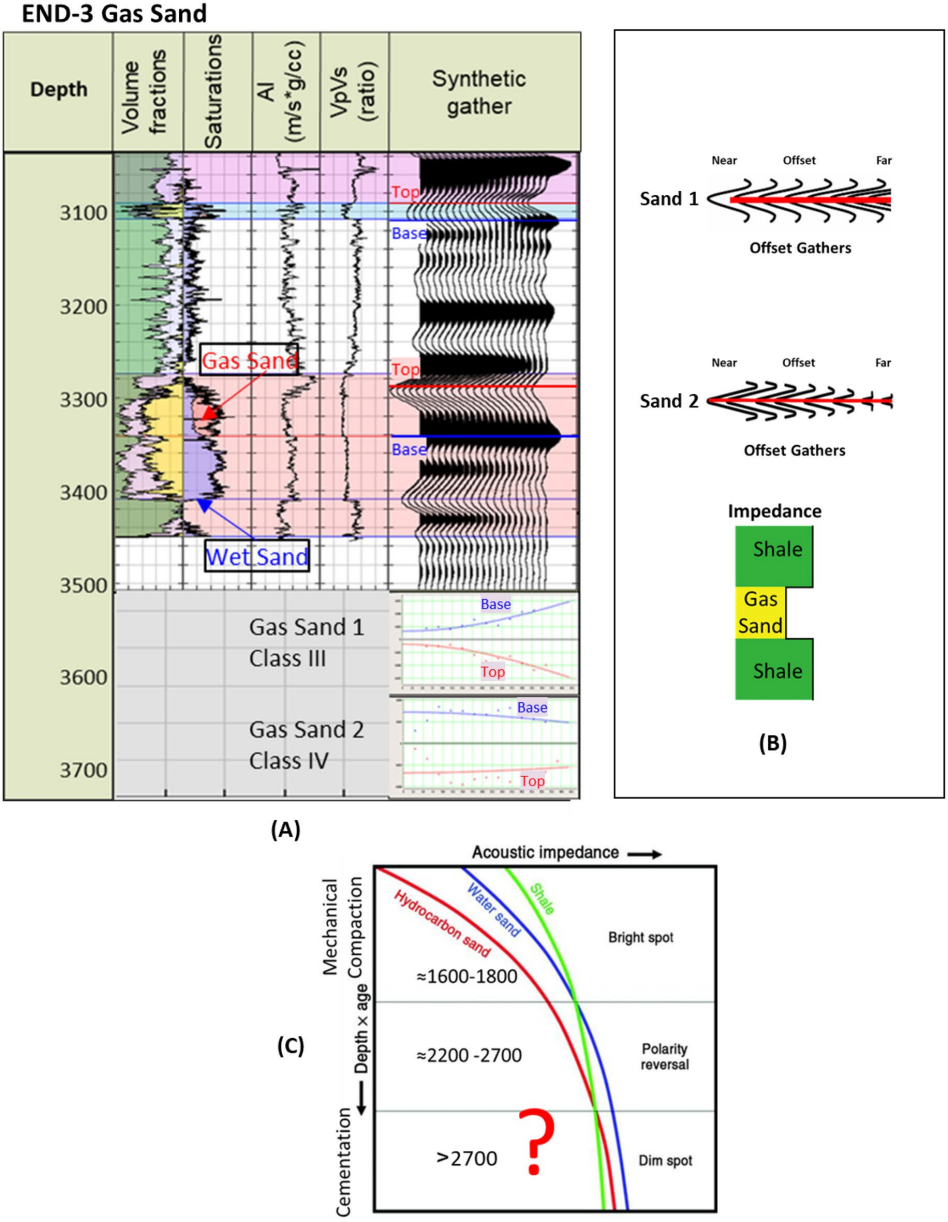
END-3 well Late Miocene gas sands 1&2, (**A**) Synthetic gather analysis shows AVO classes III & IV respectively, (**B**) Schematic form shows the impedance and offset gathers of gas sands 1&2, (**C**) Acoustic impedance trends model of gas sands 1&2 that do not follow the impedance trends.

The END-3 well (Fig. 10) penetrates the Late Miocene Messinian–Qawasim gas sand reservoirs at depths of 3100 and 3280 m for sand-1 and sand-2, respectively (Fig. 10A). The gas sand-1 and gas sand-2 that the gas sand has a significantly lower AI than the overburden shale (Fig. 10A (AI log) and B). The Qawasim gas sand-1 synthetic offset gathers indicate that the AVO behavior is gas sand Class III, starting with a bright negative amplitude (Trough) at the near offsets which increases toward the far offsets. In contrast, the Qawasim gas sand-2 behavior is AVO gas sand Class IV, with a bright negative amplitude (Trough) at the near offsets and the amplitude decreasing toward the far offsets (Fig. 10A and B).

Two wells used to evaluate the Early Miocene section, El Fayrouz-1 well to the west of the study area within the Nile Delta Basin and the Tanin-1 well to the east within the Levant Basin (Figs. 1 and 3).

**Figure 11:**
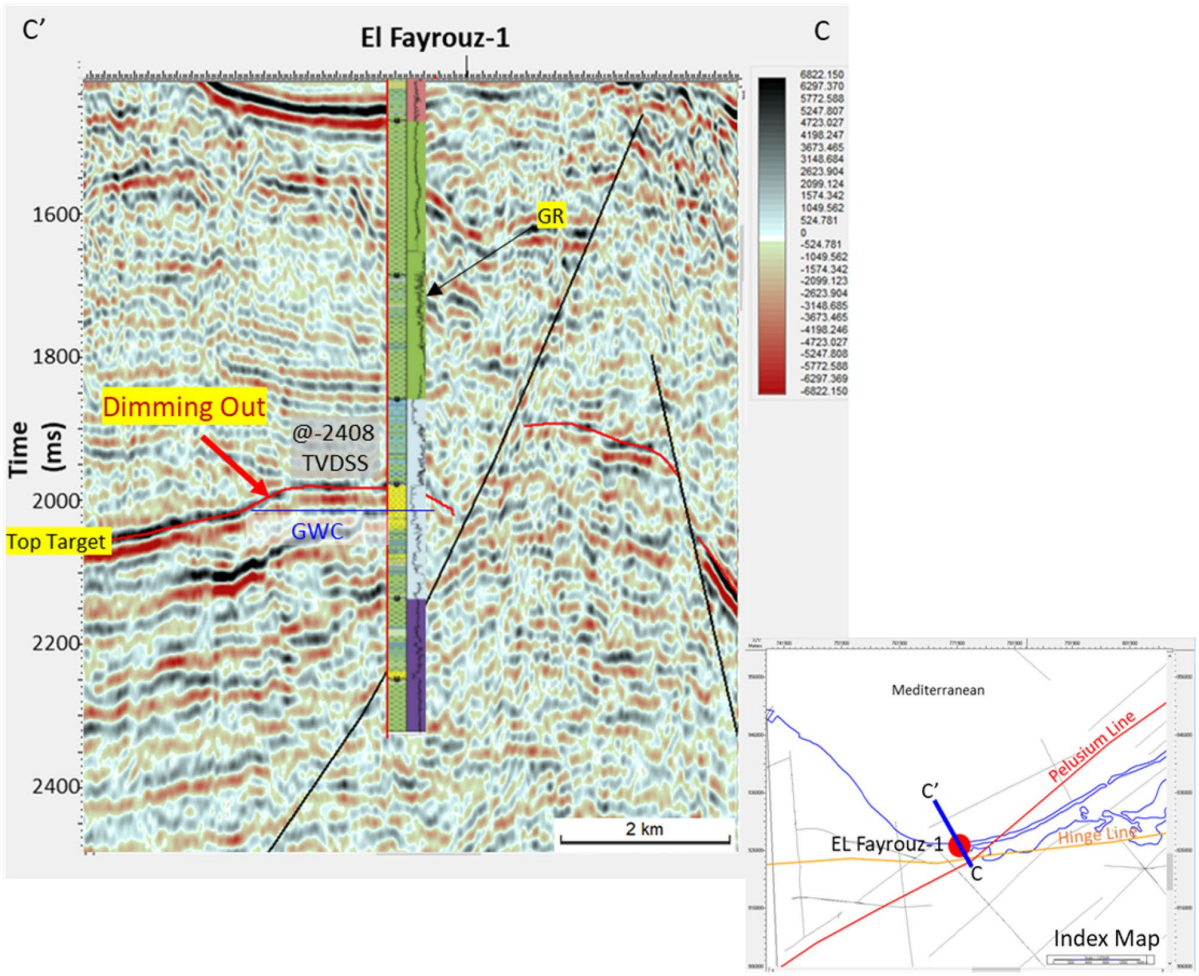
CC’ 2D seismic section as shown in Fig. 1, shows EL-Fayrouz-1 Well Lower Miocene target depth at 2408 mTVDSS that characterized by dim amplitude above the Gas Water Contact (GWC) @ 2437 mTVDSS, while the water leg characterized by bright amplitude event.

**Figure 12:**
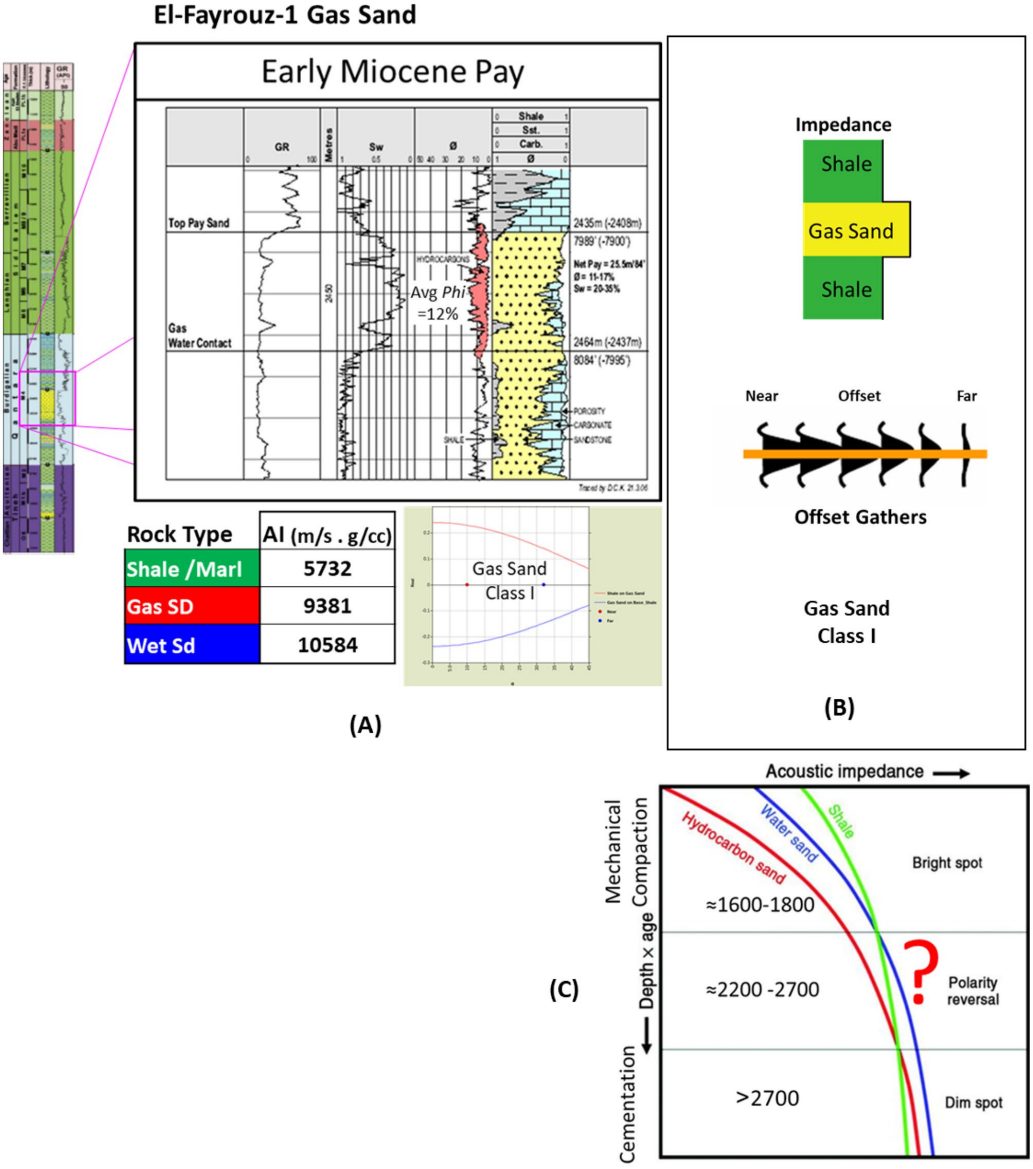
(**A**) EL- Fayrouz-1 Well Early Miocene Burdigalian gas sand calculated AI and AVO point model analysis is AVO class I, (**B**) Schematic form shows the impedance and offset gathers of gas sand, (**C**) Acoustic impedance trends model of El-Fayrouz-1 gas sand (calcareous cemented reservoir) doesn’t follow the impedance trends.

The Nile Delta Early Miocene El Fayrouz-1 well tested a dim seismic amplitude of gas sand within the Early Miocene structure, down-dip of the Hinge Zone and Pelusium Line (Figs. 1 and 10), the Burdigalian top target depth at 2408 m TVDSS (Figs. 10 and 11A). The reservoir was described as calcareous sandstone (highly cemented) and the overburden rock was described as calcareous shale, graded to Marl (Fig. 12A).

The AI of the gas sand is around 9381 (g/cc.m/s), which is significantly higher than the overburdened rock of 5732 (g/cc.m/s) (Fig. 12A). The AVO point model shows behavior being gas sand Class I (dim-out) at a depth of 2435 m. The AVO signature starts with a bright positive amplitude (Peak) at the near offset which then decreases toward the far offset (Fig. 12B) as the AVO point model shows in (Fig. 12A).The AI of El Fayrouz-1 Early Miocene Qantara sandstone at a depth 2435 m should behave the polarity reversal phase stage, while the actual response follows the dimout amplitude stage3 and doesn’t follow the expected AI trends and seismic behavior with a depth of stage 2, because of the highly calcareous cemented reservoir (Fig. 12C).

**Figure 13:**
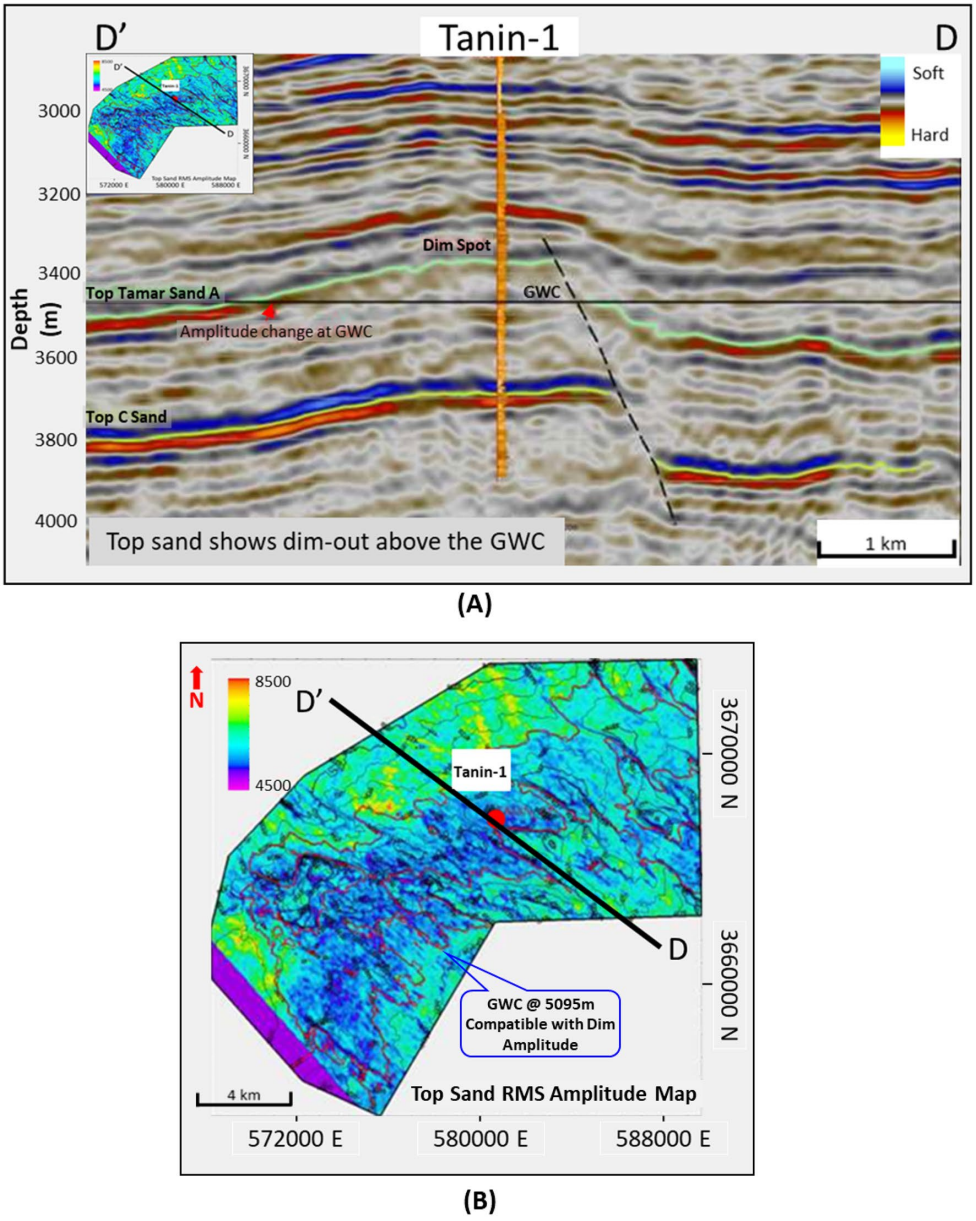
(**A**) DD’ Seismic section across Tanin Early Miocene (Burdigalian) structure, where the top Tamar sand-A characterized by dim amplitude above the GWC at 5095 mTVDSS as the spill point of the structure, (**B**) Amplitude map overlayed by structure contour lines which shows the good match of the dim amplitude with the structure spill (Karish and Tanin field development plan^77^). Created using kingdom 2015 software, https://kingdom.ihs.com/.

**Figure 14:**
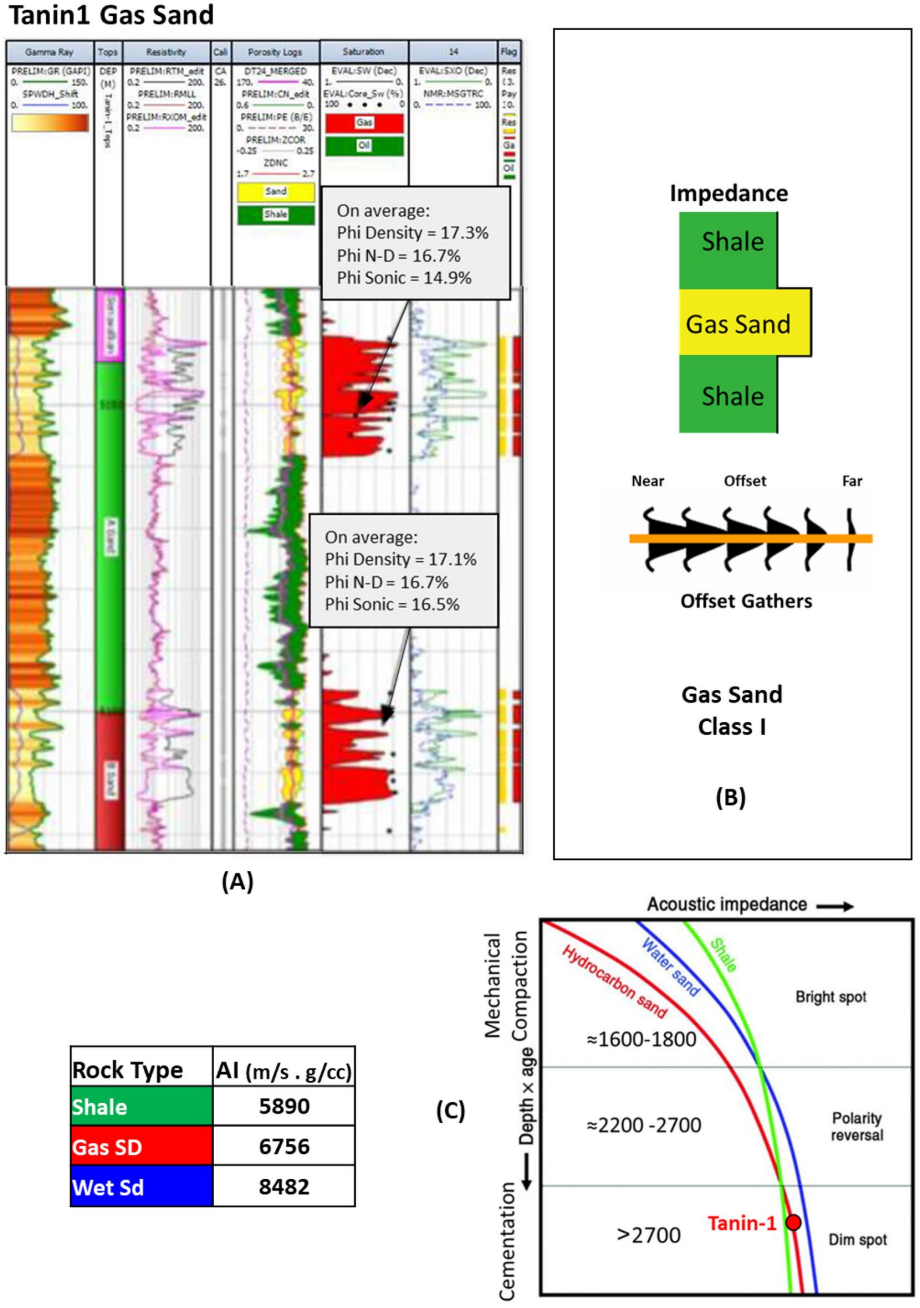
(**A**) Tanin-1 well Early Miocene gas sand evaluation at depth 3310 m, (**B**) Schematic form shows the impedance and offset gathers of gas sand, (**C**) Acoustic impedance trends model of Tanin-1 wet & gas sand that plotted within the dim spot zone.

**Figure 15:**
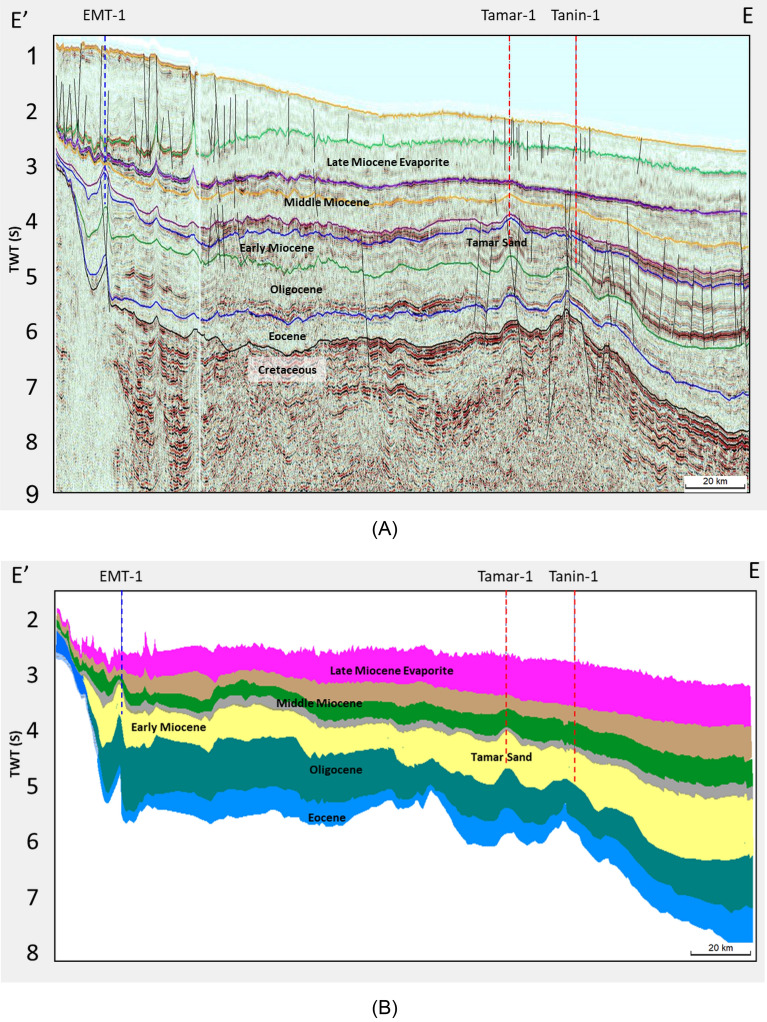
(**A**) EE’ Seismic section, (**B**) EE’ Stratigraphic section passing by EMT-1, Tamar-1, and Tanin-1 wells shows the Early Miocene target reservoir extension.

On the other hand, within the Levant Basin eastward the Early Miocene discovery Tanin-1 well penetrates the Oligo-Miocene stacked marine basin floor fan sands (Tamar sand) at depth of 3310 m, which was penetrated by Tamar and Leviathan wells (Figs. 1, 3, 13 and 15). The seismic data indicate that the gas sand is dim-out in amplitude above the Gas Water Contact (GWC) as shown in the seismic section passing by the Tanin-1 well, also the RMS amplitude map of the top reservoir (Figs. 13 and 14)77.

The AI calculated for the gas sand around 7550 (g/cc.m/s) is higher than the AI for the overburden shale of 7200 (g/cc.m/s), while the wet sand AI is 8480 (g/cc.m/s) is significantly higher. In this case, the Tanin-1 gas sand seismic signature follows the expected AI depth trends for hydrocarbon saturation sands (Fig. 14C).

### Application of concept to EMT-1 well

The EMT-1 well targeted a reservoir rock in an Early Miocene (Burdigalian-Aquitanian) Syrian Arc System Phase II anticlinal structure. Also, the same stratigraphic section was drilled by the El Fayrouz-1 well within the Nile Delta Basin and similar sands were drilled by the Tanin-1 and Tamar-1 wells within the Levant Basin (Fig. 15). As outlined above, the evaluation of the nearby discoveries indicates non-unique behavior of the seismic character with depth, with factors such as depositional environment, sediment supply, and cementation type affecting the actual behavior. So, the assess of the AVO modeling and AI responses of EMT-1 are mandatory to describe the seismic signature characteristics with depth for the Early Miocene reservoir section within the study area.

#### Pre-drilling evaluation and concept

The PSDM seismic data used to evaluate the EMT-1 well target horizon exhibits a seismic amplitude dim-out over the structure with conformance occurring at the structural spill depth of 3530 m TVDSS (Fig. 7A). The seismic amplitude dim-out was expected to be affected by cementation or diagenesis as it deeper than the target in El Fayrouz-1 well. The top target depth for the EMT-1 well was around 3300 m TVDSS (Fig. 7A). According to the seismic signature and the AI trends with depth, the target is located within the dimout phase zone, where the gas sand could be either AVO Class I.

**Figure 16:**
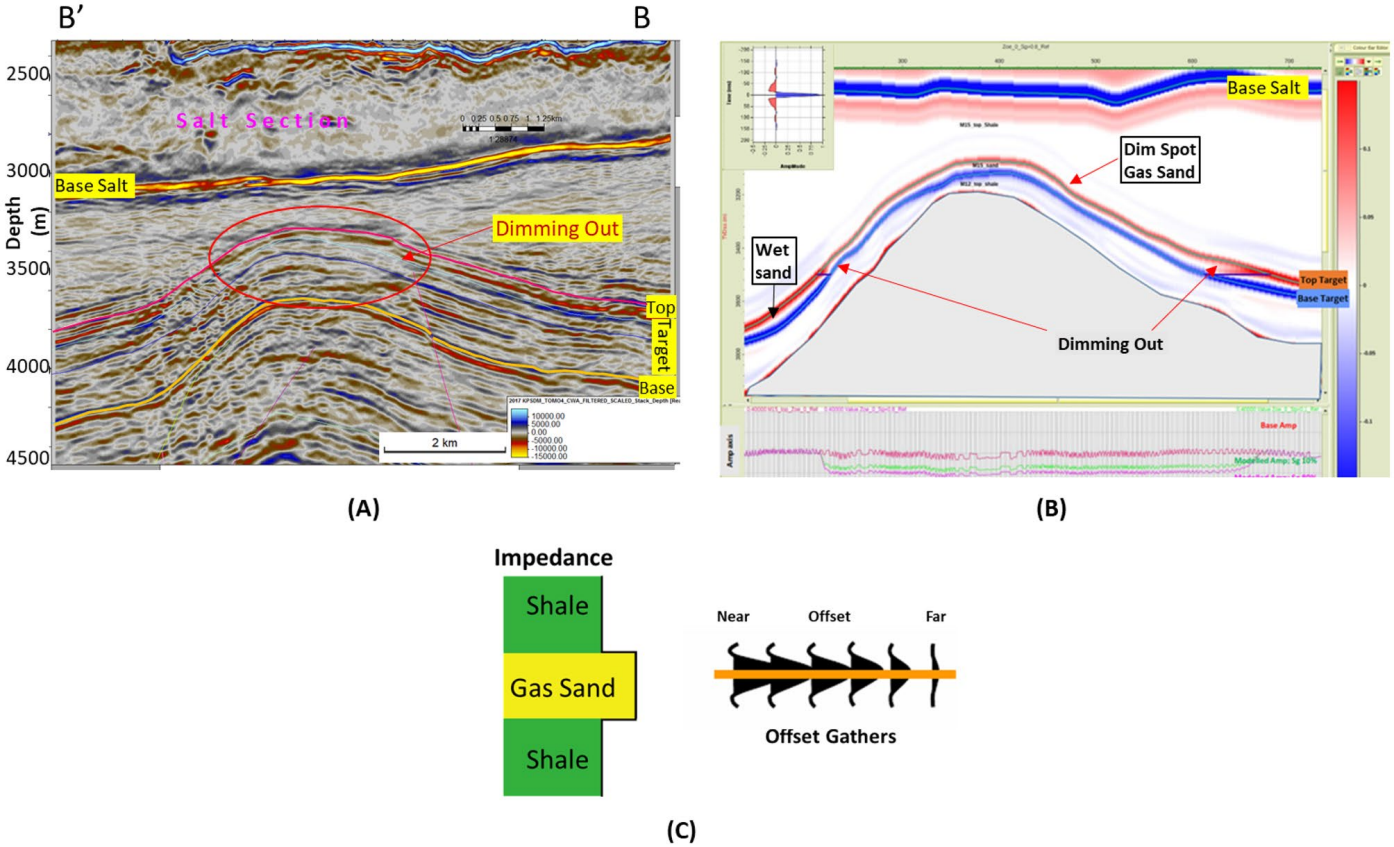
(**A**) EMT-1 pre-drilling seismic section BB’ shows the dimming out amplitude at the top of the structure, (**B**) 2D synthetic model for a single layer within EMT-1 structure using the nearby wells petrophysical parameters *(Background shale parameters (Clay-rich): Vp* = *2120 m/s; Rho 2.24 g/cc, Wet sand parameters (Quartz* = *0.9 & Clay* = *0.1): Vp* = *3300 m/s; Rho 2.33 g/cc , SW* = *100%, Gas sand parameters (Quartz* = *0.9 & Clay* = *0.1): Vp* = *2900 m/s; Rho 2.1 g/cc , SW* = *20% ),* (C) Schematic form shows the impedance and offset gathers of expected gas sand.

A 2D synthetic seismic forward model for the EMT structure was constructed for the gas sand case using data available from the nearby El Fayrouz-1 and Tanin-1 wells. The result showed an amplitude dim-out supporting the evaluation of gas within the EMT reservoir (Fig. 16). According to this model, a gas sand is expected to have a higher AI than the overburden shale and exhibit AVO Class I gas sand behavior. The water-filled sand is expected to have an even higher AI than that of the gas sand and overburden.

#### Post-drilling evaluation

**Figure 17:**
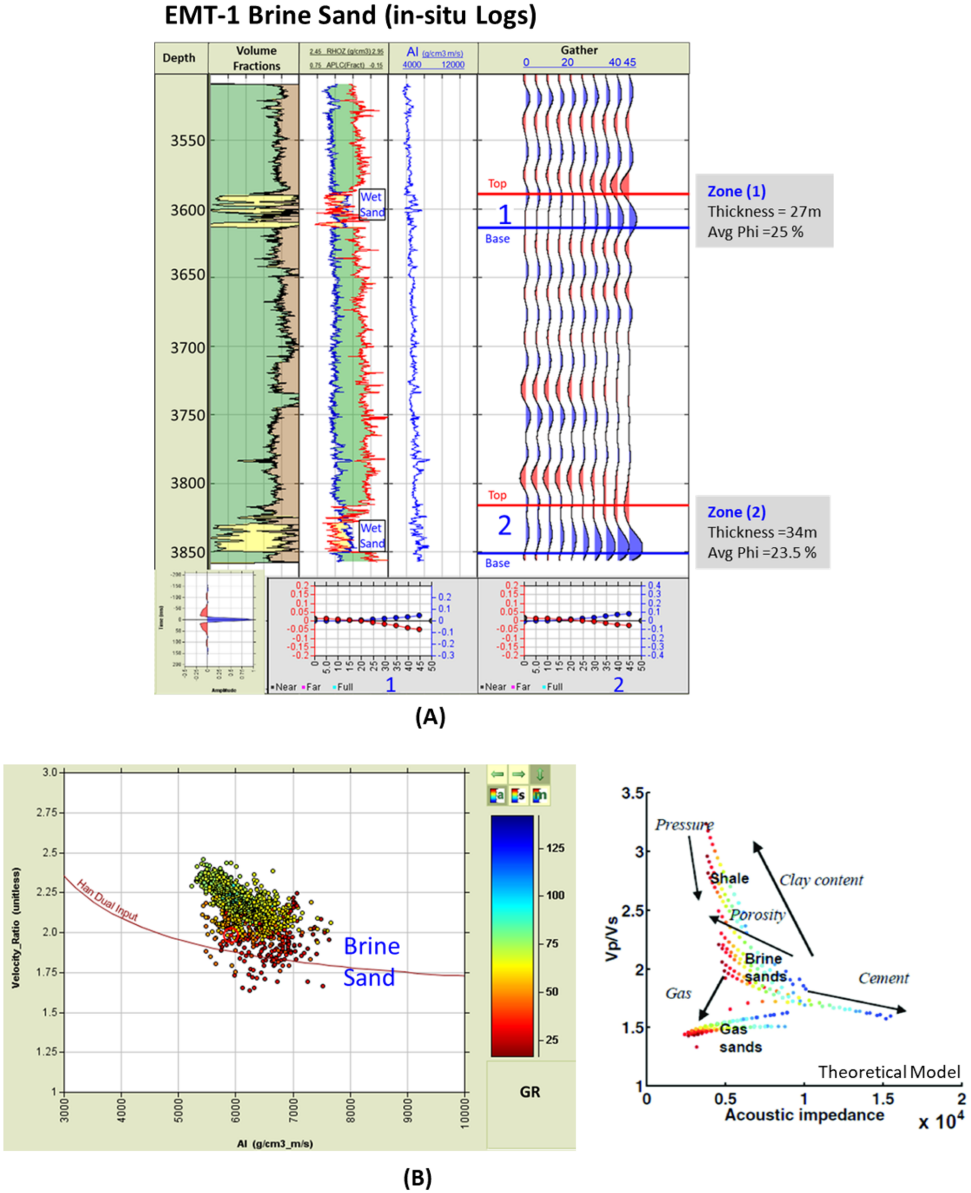
EMT-1 well Early Miocene wet sand 1&2 at depths 3580 m and 3817 m, (**A**) Synthetic gather analysis shows AVO class I or IIp as the seismic signature started by peak and reversed to trough after incident angle 20°, (**B**) In-situ data AI/ Velocity Ratio (Vp/Vs) Cross-plot that shows that the smallest (Gama Ray) GR values is represented by ideal brine sand as the theoretical model shows.

As targeted, the EMT-1 well encountered two wet sand bodies within the Aquitanian (Fig. 17). The penetrated sand reservoir is not as thick as the Basin Floor Fan sands that exist within the Levant Basin. The reservoir has good quality petrophysical parameters with an average porosity of 24% (Fig. 17A). The first sand body (Zone-1) thickness is 27 m with an average porosity of 25% (as the Neutron-Density logs show in (Fig. 17A) and average AI of ~ 6250 (g/cc.m/s) at 3580 m TVDSS (Fig. 17A, Zone-1) that compares to the average AI for the overburden of ~ 5800 (g/cc.m/s). The second sand body (Zone-2) is 34 m thick with an average porosity of 23.5% and average AI of ~ 6700 (g/cc.m/s) at 3817 m TVDSS (Fig. 17A, Zone-2). The average AI for the shale overlying the second sand is ~ 6400 (g/cc.m/s). By comparison, the petrophysical parameters for the EMT-1 sands are better than those of the El Fayrouz-1 and Tanin-1 wells (Figs. 12, 14 and 17).

**Figure 18:**
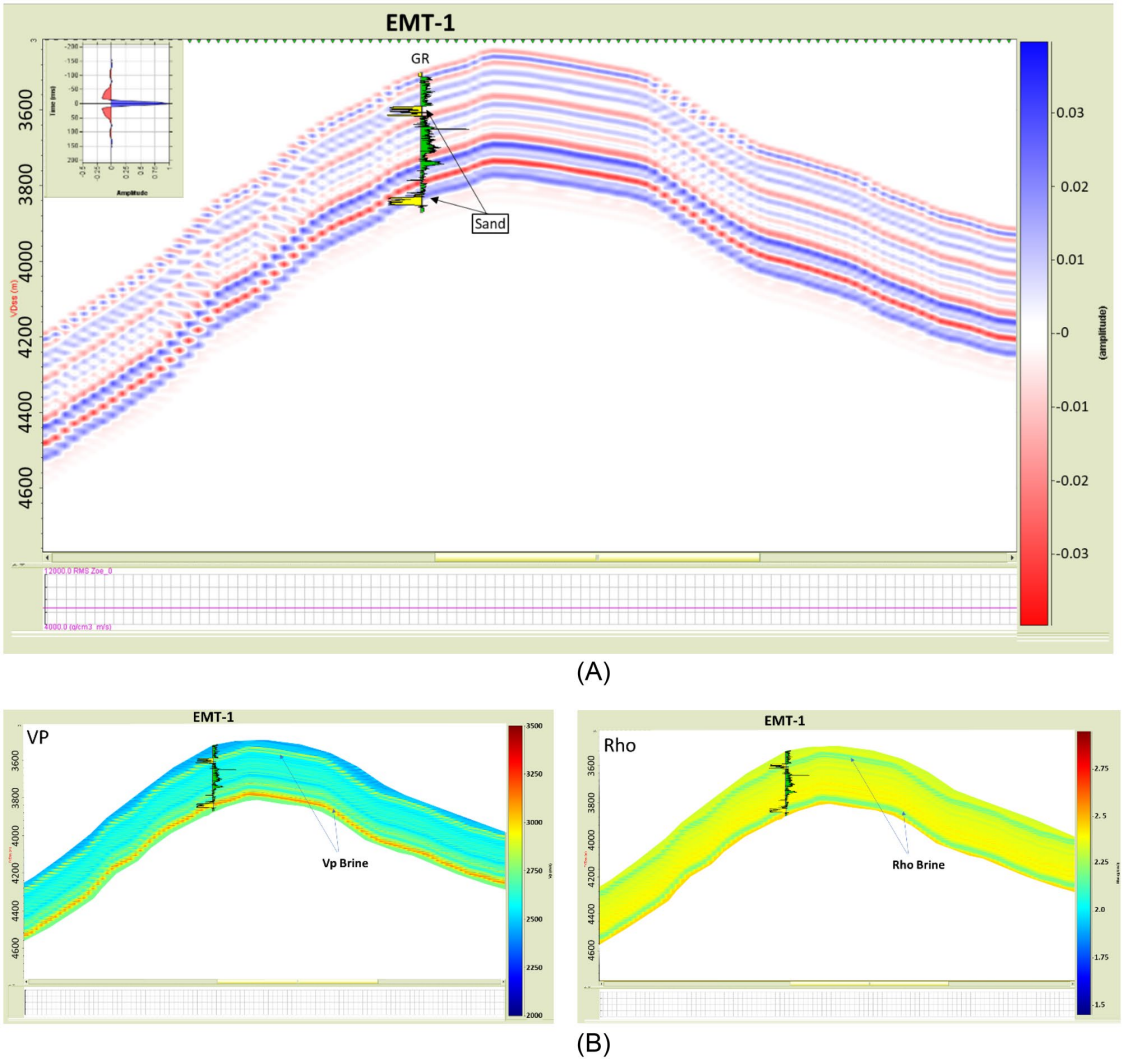
(**A**) 2D Synthetic model for EMT structure, using the EMT-1 well in-situ data, (**B**) Geophysical models using EMT-1 well in-situ data velocity Vp and density Rho.

The synthetic angles gather for the EMT-1 well were created using in-situ data. The result of the AVO analysis is Class I or Class IIp for the wet sand since the seismic signature starts with a peak and switches to a trough at angles greater than 20 degrees (Fig. 17A). The AI versus VP/VS cross plot, as colored by Gamma Ray (GR), clearly indicates a wet sand as the theoretical model cross-plot shows (Fig. 17B). A 2D synthetic seismic model for the structure constructed using the EMT-1 well in-situ brine sand data shows continuity of the seismic reflectivity without amplitude change at the crest and flanks of the structure (Fig. 18A). Also, the resultant velocity and density models are smoothed and compatible with the in-situ data of EMT-1 well (Fig. 18B).

#### Fluid replacement model (EMT-1 Gas Case)

**Figure 19:**
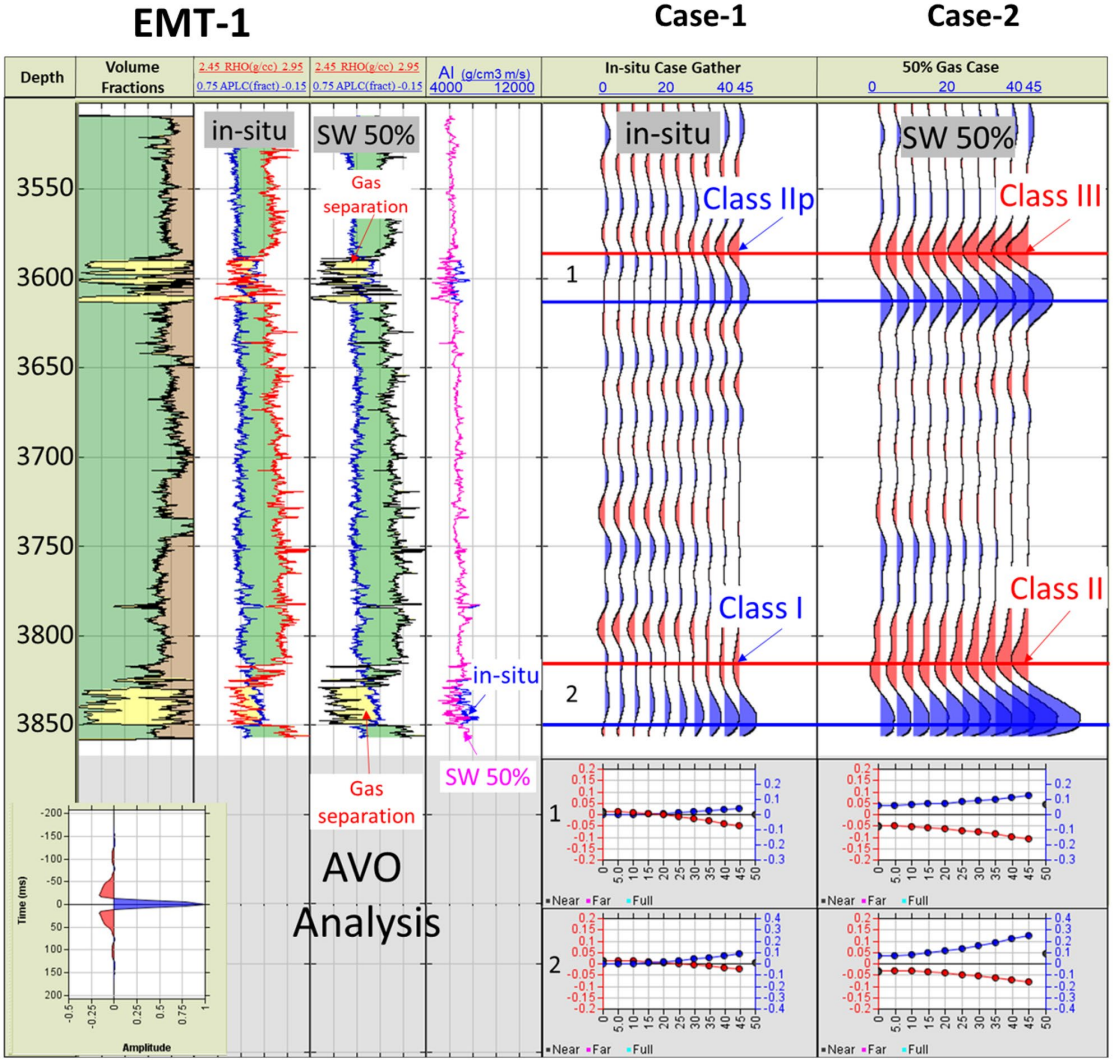
EMT-1 well Early Miocene reservoir synthetic gather, and AVO analysis for the in-situ logs case-1 and the fluid substituted logs SW 50% case-2, Synthetic Gather AVO analysis shows, case-1 wet sands zones 1&2 are class I or IIp, while case-2 is the fluid substituted case (SW 50%) for the zones 1&2 are class II or III.

**Figure 20:**
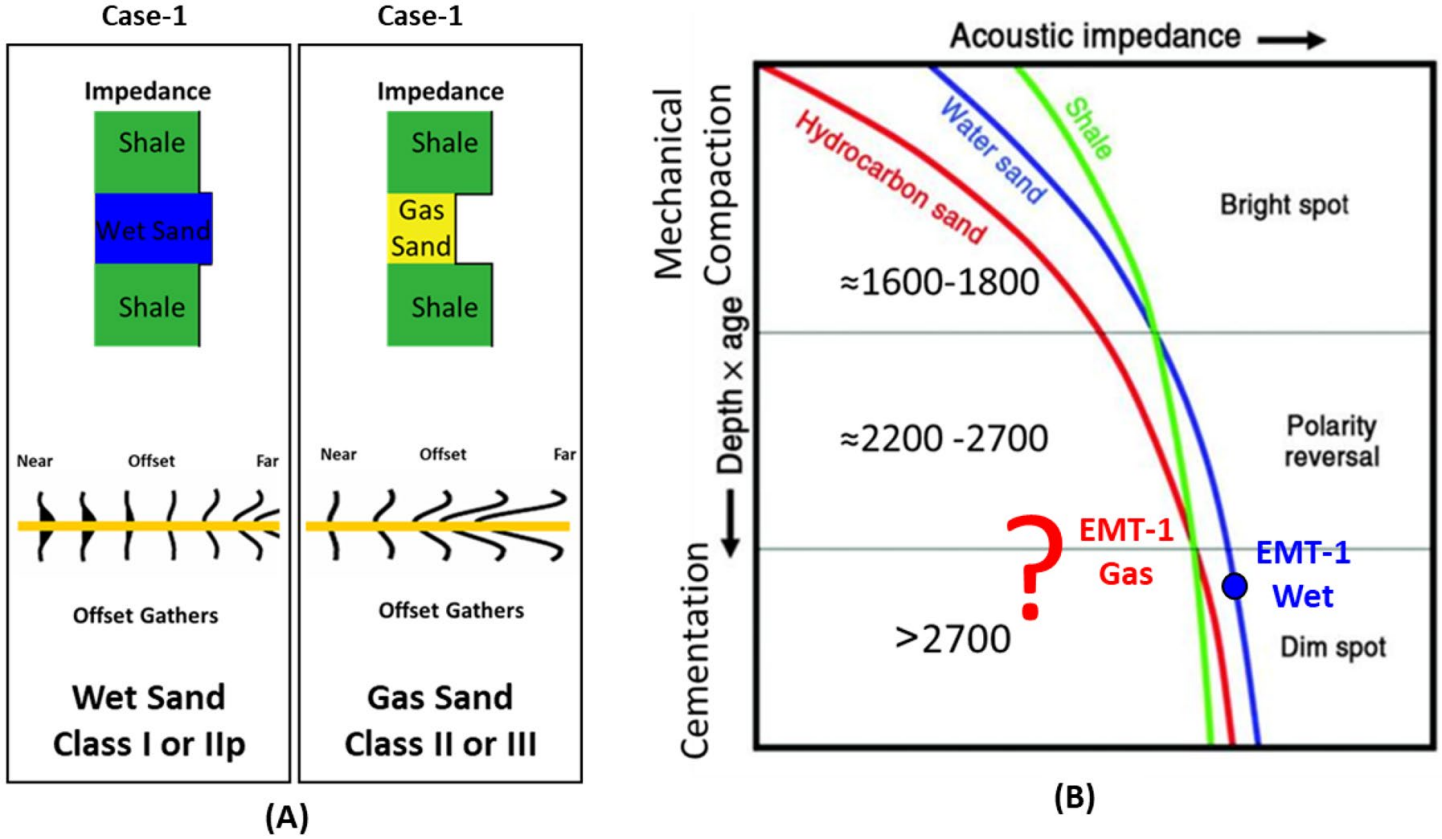
EMT-1 well Early Miocene reservoir; the in-situ logs case-1 and the fluid substituted logs SW 50% case-2, (**A**) Schematic form shows the impedance and offset gathers of wet sand (ins-situ case) and the gas sand (SW 50% case), (**B**) Acoustic impedance trends model of case-1 wet sand that plotted within the dim spot zone and case-2 gas sand (SW 50% case) which do not follow the acoustic impedance trends model.

A fluid replacement was carried out for the EMT-1 well logs (Fig. 19), to understand the gas sand seismic behavior assuming a gas saturation (SG) of 50% and water saturation (SW) of 50% for the EMT-1 case-2. The effect of gas separation is obvious on the density-neutron plot and the gas sand AI is lower than both the overburden shale and the brine sand as (Figs. 19 and 20A). The AI of the wet sandstone versus depth trend plot exists within the dim-out (Dim Spot) Stage 3 zone, while the gas sand falls within the polarity reversal Stage 2 zone (Figs. 20B and 22).

**Figure 21:**
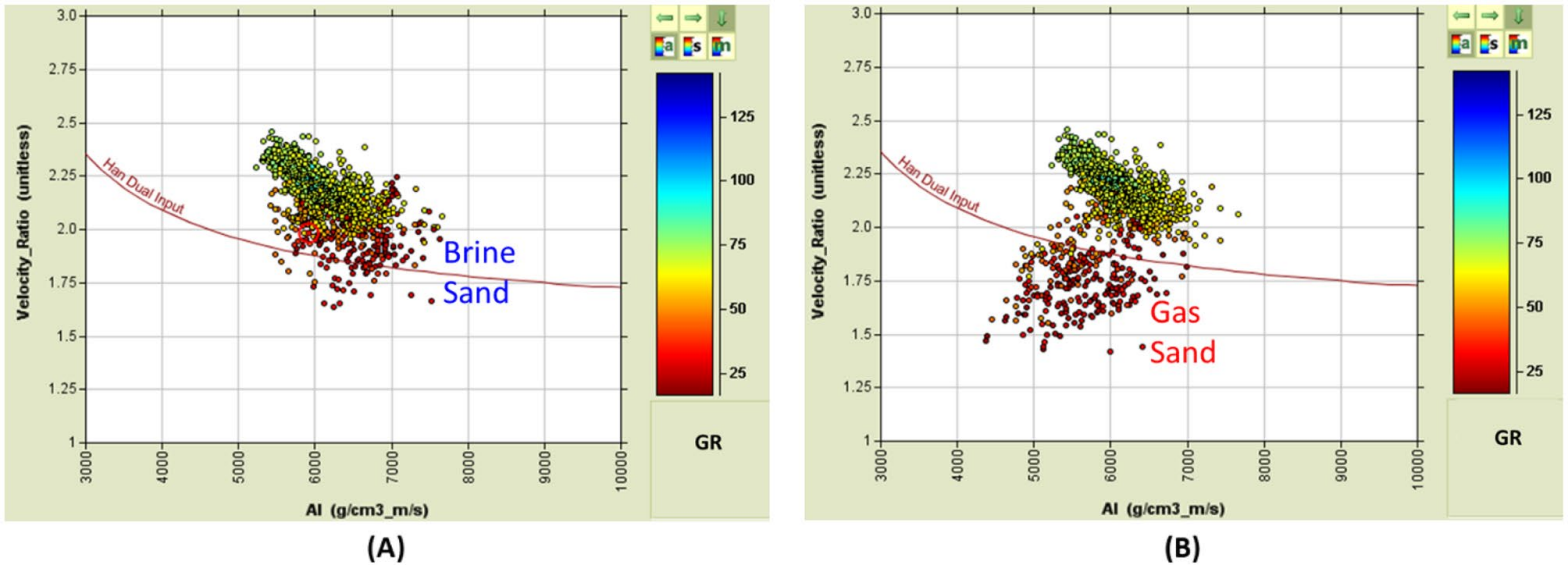
EMT-1 well acoustic impedance versus velocity ratio (VP/VS) Cross-plot, (**A**) In-situ log data AI/ (Vp/Vs) Cross-plot shows that the smallest GR values is represented by ideal brine sand, (**B**) Fluid substituted data, SW 50% data AI/VPVS Cross—plot shows that the smallest GR values is represented by ideal gas sand.

The synthetic gathers for the 50% gas-substituted sandstone (EMT-1 case-2) shows AVO analysis gas sand Class II or III, wherein the top reflector starts with a bright or slightly bright trough at the near angles with the amplitude increasing toward the far angles (Fig. 19). Figure 21 shows the AI versus VP/VS cross plot, as colored by GR, for EMT-1 both cases, the in-situ wet sandstone and the 50% gas-substituted logs, which clearly display the separation between the gas sand and the wet background trend (Fig. 21B).

## Discussion

The AI trends with depth of the Late Miocene reservoirs of the Nile Delta wells END-1, END-2, and END-3 don’t exhibit a unique seismic response or AI vs depth behavior, as END-1 and END-2 follow the expected AI trends and the AVO response of Class II and class IIp as the reservoir affected by the chemical compaction at depths of the Stage 2 between 2580 And 2630 m (Figs. 8 and 9). The AI behaviors of the END-3 Qawasim sands within the deeper section do not follow the expected AI trends and behavior with depth (Fig. 10), because the reservoir sands are preserved with high-quality reservoir with significant low AI than the overburden shale and wasn’t affected by cementation at depth Stage 3 more than 3000 m, with AVO Class III and IV which the response of the shallow section of Stage 1 (Figs. 6 and 10). This demonstrates changes in AI with the depth do not exhibit unique behavior.

**Figure 22:**
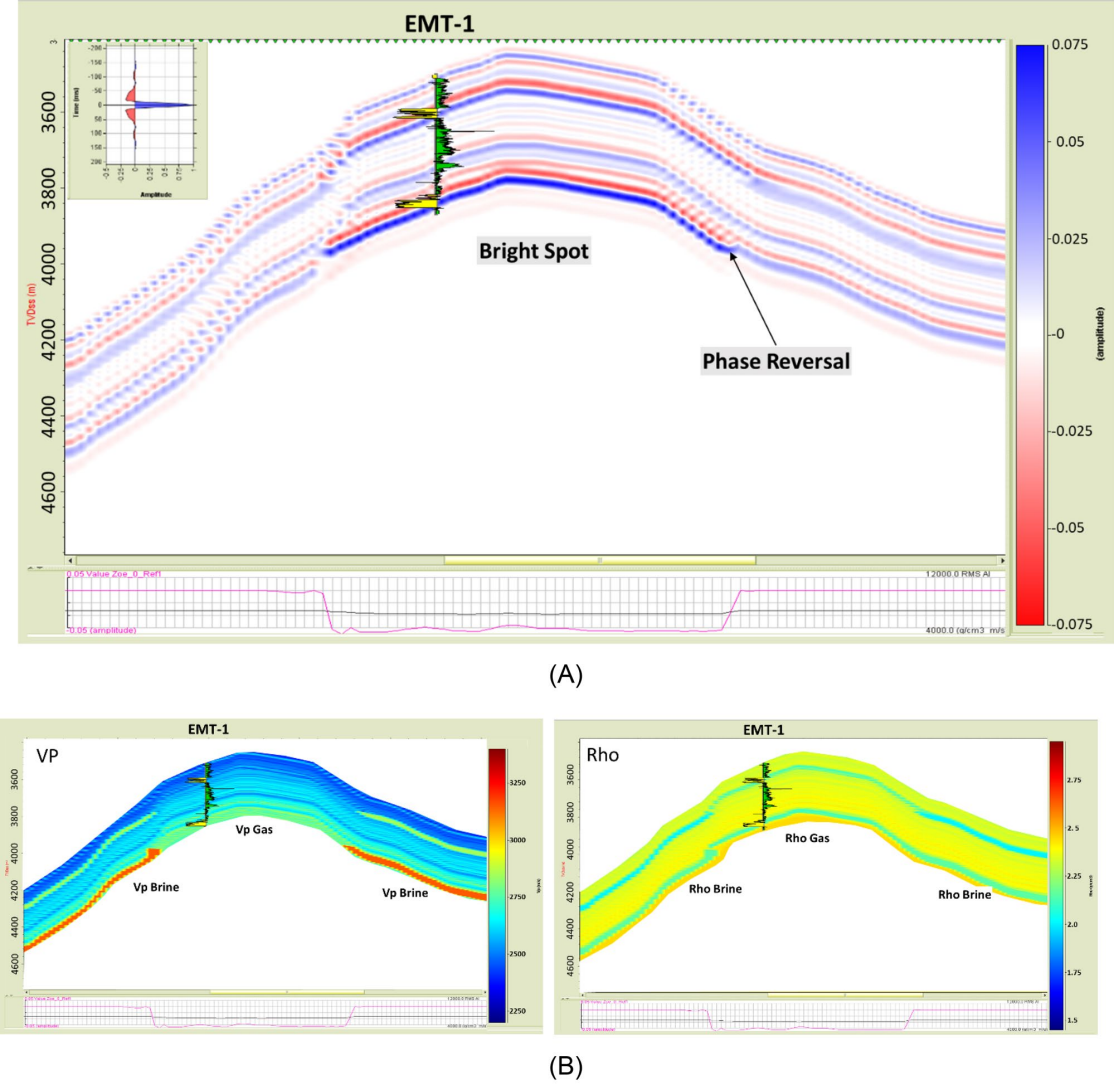
(**A**) 2D Synthetic model for EMT structure using EMT-1 well with 50% SW log data, (**B**) Geophysical models using EMT-1 well with 50% SW log data velocity Vp and density Rho.

The Early Miocene reservoir of the Nile Delta well El Fayrouz-1 well highly calcareous cemented reservoir sand at a depth of 2435 m does not follow the expected trend of AI with depth. The AVO response of a gas sand Class I at depth of the Stage 2 (Fig. 12). The Levant Basin Tanin-1 well reservoir gas sand follows the expected trend of AI with depth and the AVO response is that of a gas sand Class I at depth of 3310 m of the Stage 3 (Fig. 13). At this depth, the Tanin-1 reservoir sand should also be highly cemented. Moreover, the forward model for EMT-1 structure for gas sandstone shows dim-out amplitude (Fig. 16B). This makes the evaluation of the seismic data within the study area critically important since amplitude dim-out within the Early Miocene anticlinal structure within the study area (EMT) is observed at depth 3300 m (Fig. 22).

**Figure 23:**
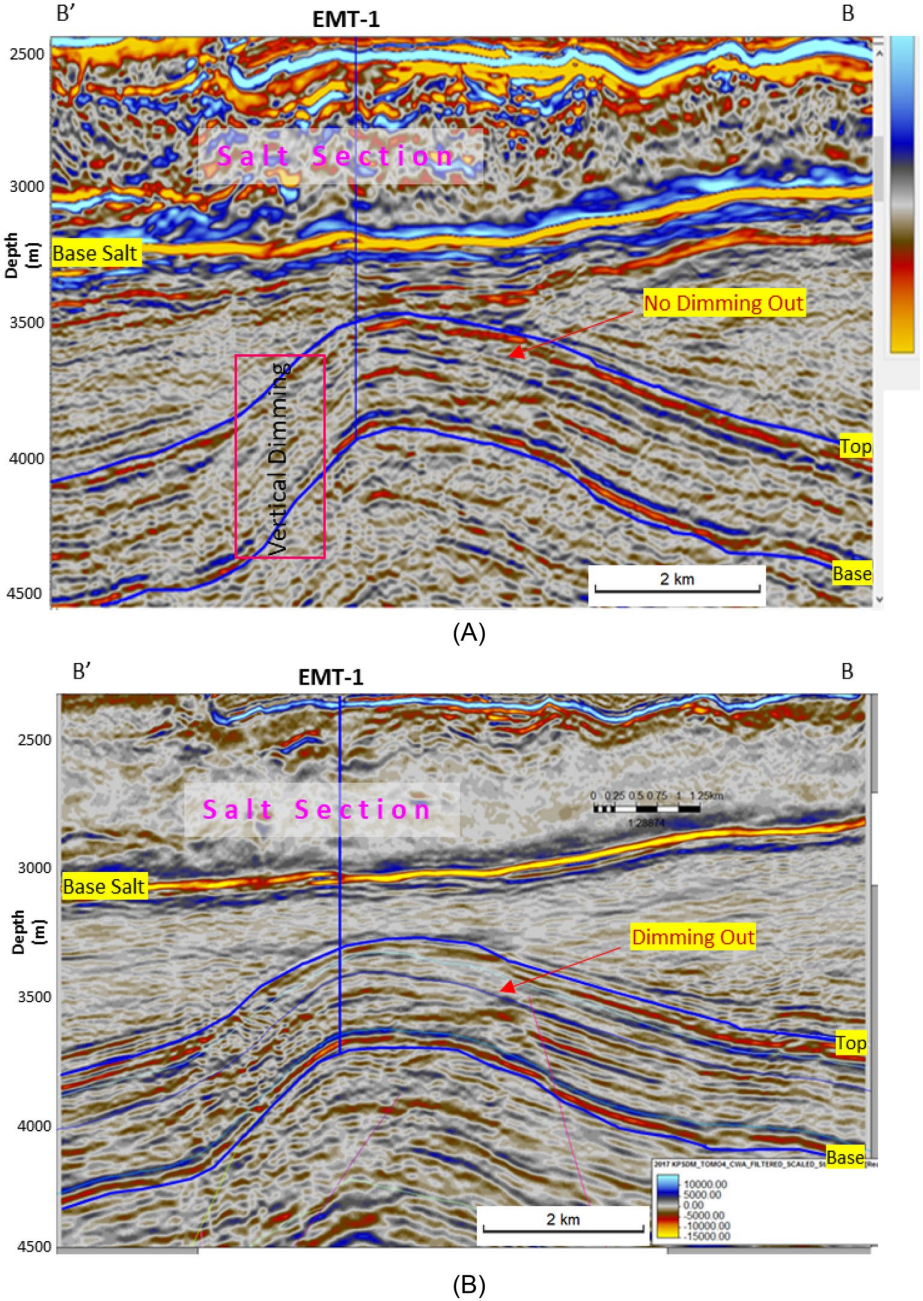
EMT Miocene structure seismic section, comparing (**A**) The result of the Post drilling reprocessed seismic section with (**B**) The pre- drilling seismic section.

The seismic synthetic modeling for the Early Miocene reservoir of the EMT-1 in-situ data (water filled sand) shows that the reservoir AI is higher than the overburden shale and follow the expected AI trends and behavior of the AVO response Class I of the Stage 3 at depths 2830 m and 3067 m for wet sand 1 and 2 at respectively (Fig. 20B). Also, the 2D synthetic seismic model (Fig. 18) shows, there is no amplitude dimming above the structural spill point as the original legacy 3D seismic data demonstrate (Fig. 7 and 16). To understand the cause of the observed amplitude dim-out in the original seismic analysis, the 3D seismic data was reprocessed using a new velocity model based on the EMT-1 data. The resultant reprocessed 3D seismic data behavior differed from the original interpretation, as the dim-out and spill-point conformance were no longer observed at EMT-1 structure (Fig. 23).

EMT-1 fluid substitution gas sand shows ideal gas separation signature on the density-neutron logs (Fig. 19). The AI of the gas sand and AVO seismic response is gas sand Class II or III at depths 2830 m and 3067 m don’t follow the expected trends of the Stage 3 (Fig. 20B). The 2D synthetic seismic model for the structure was constructed using the 50% gas-substituted EMT-1 well as the top gas sand above the spill point of the structure represented by soft event “trough” and beneath the spill point the in-situ logs (wet sand) used which represented by hard event “peak”. The results indicate a phase reversal above the structural spill point at the water leg, as represented by the peak, which becomes soft and converted to a trough for the gas sandstone. This scenario shows that the gas sand clearly exhibits a bright spot, which behaves the opposite seismic response of the pre-drill concept (Fig. 22).

## Conclusions

- The Eastern Mediterranean Miocene Section is a principal source for both discovered and prospective gas within the Nile Delta Cone and Levant Basin.
- The Miocene sections consist of different sedimentary environments comprised of turbidite slope deposits, basin floor fans, and channel sands, and capped by the Messinian Salinity Crisis evaporites.
- A study of rock properties for the different Miocene reservoirs and sections revealed the possibility of heterogeneity-induced ambiguity within the seismic response (Non-unique AI behavior observed as a function of depth).
- This study concluded that the Early Miocene reservoir's dim-out amplitude of the pre-drill EMT-1 seismic data is not due to the presence of gas.
- Readjust the processing velocity model of the Messinian salt confirms that there is no change of the seismic amplitude through the EMT-1 structure, as well as the post-drill in-situ well logs data forward model of the wet sandstone at depths 3580 and 3817.
- The EMT-1 fluid replacement model assumes the gas saturation SG = 50% and is characterized by a bright, soft “trough” of AVO gas sandstone Class II or III.
- Seismic data DHI features are not necessarily related to hydrocarbon accumulation.
- Seismic QI analysis could be useful in reducing seismic processing uncertainty.
- AVO, seismic signature, and AI trend with depth don't have a unique solution for the different Miocene reservoirs.
- Proper seismic acquisition planning and appropriate processing techniques are vital tools for successful seismic interpretation with the ultimate goal of hydrocarbon discovery.

## Data Availability

The data that support the findings of this study are available from [the Egyptian General Petroleum Corporation] but restrictions apply to the availability of these data, which were used under license for the current study, and so are not publicly available. Data are however available from the authors upon reasonable request and with permission of [the Egyptian General Petroleum Corporation].
